# Controlling Exsolution Dynamics in High‐Entropy Oxides for Highly Active and Selective Acetylene Semi‐Hydrogenation

**DOI:** 10.1002/anie.9920205

**Published:** 2026-05-15

**Authors:** Hailing Yu, Caiqi Wang, Kevin M. Siniard, Qingju Wang, Yuanpeng Zhang, J. Anibal Boscoboinik, Xiao Tong, Eliseo Perez Gomez, Shuai Yuan, Arun S. Asundi, Oliver Mueller, Murillo Longo Martins, Yongqiang Cheng, Michael Richard Koehler, De‐en Jiang, Zili Wu, Zhenzhen Yang, Sheng Dai

**Affiliations:** ^1^ Department of Chemistry Institute for Advanced Materials and Manufacturing University of Tennessee, Knoxville Knoxville Tennessee USA; ^2^ Chemical Sciences Division Oak Ridge National Laboratory Oak Ridge Tennessee USA; ^3^ Neutron Scattering Division Oak Ridge National Laboratory Oak Ridge Tennessee USA; ^4^ Center for Functional Nanomaterials Brookhaven National Laboratory Upton New York USA; ^5^ Department of Chemical and Biomolecular Engineering Vanderbilt University Nashville Tennessee USA; ^6^ Stanford Synchrotron Radiation Lightsource SLAC National Accelerator Laboratory Menlo Park California USA; ^7^ Institute For Advanced Materials and Manufacturing Diffraction Facility University of Tennessee Knoxville Tennessee USA

**Keywords:** acetylene semi‐hydrogenation, heterogeneous catalysis, high entropy oxides, lattice engineering, metal exsolution

## Abstract

Exsolution‐derived catalysts feature robust metal–support interactions that enhance catalytic performance; yet achieving precise control over exsolution dynamics in multicomponent oxides remains challenging. In this study, we demonstrate that exsolution behavior in high‐entropy oxides (HEOs) can be rationally tuned through coupled lattice‐ and valence‐engineering to create a highly active and selective catalyst for acetylene semi‐hydrogenation. Incorporation of Li^+^ into a rock salt‐structured HEO (LiNiMgCuZnCoO_x_ and LiHEO) induces local lattice distortion, generates oxygen vacancies, and partially oxidizes Co sites from Co^2+^ to Co^3+^, collectively modulating local charge redistribution. This strategy enables facilitated Cu nanoparticle exsolution and alters the exsolution sequence from Cu^0^ > Ni^0^ > Co^0^ in pristine HEO to Cu^0^ > Co^0^ > Ni^0^ in the LiHEO. The resulting catalyst via controlled exsolution exhibits superior activity and ethylene selectivity, outperforming state‐of‐the‐art transition metal systems. This work establishes entropy‐enabled lattice and valence engineering as a facile route to programmable exsolution for enhanced catalysis.

## Graphical abstract

Li^+^‐induced lattice distortion, valence variation, and local charge redistribution in a rock salt‐structured high‐entropy oxide enable controlled metal exsolution, altering the Cu–Ni–Co exsolution sequence and delivering a surface Cu^0^‐enriched catalyst with exceptional activity and ethylene selectivity for acetylene semi‐hydrogenation.

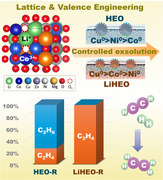

## 1 Introduction

Metal‐support interaction (MSI) is widely recognized as one of the central pillars of heterogeneous catalysis, since it not only regulates the dispersion, electronic structure, and stability of active metal species, but also governs the adsorption, activation, and subsequent transformation of reactant molecules on catalyst surfaces [1, 2, 3, 4, 5]. Recently, the exsolution of metal nanoparticles (Pd, Ru, Pt, Ni, Co, and Fe) from host oxides with diverse structures under reductive conditions has emerged as a powerful strategy to constructing MSI in heterogeneous catalysts relevant to diverse catalytic processes (e.g. dry reforming of methane (DRM) and CO_2_ reduction) [6, 7, 8, 9, 10, 11, 12, 13, 14]. Exsolution is a process in which metal cations migrate from the bulk lattice to the surface under reductive environments, forming finely dispersed and strongly anchored metallic nanoparticles (NPs) [15, 16]. In this context, it represents a dynamic manifestation of MSI, as the host lattice not only dictates nanoparticle nucleation and stabilization but also continuously modulates their electronic and structural states during catalysis. Compared with conventional supported metal catalyst prepared by impregnation or deposition, exsolved metal NPs often provide uniform particle size distributions and reinforced MSI, thereby enhancing catalytic performance. For instance, Pd NPs exsolved from LaFe_0.57_Co_0.38_Pd_0.05_O_3_ (Pd‐perovskite) maintained high catalytic stability during aging for automotive emission control, in contrast to the rapid deactivation observed in the Pd‐impregnated γ‐Al_2_O_3_ catalyst [6]. The reductive exsolution of metallic Ru from fluorite‐type Sm_2_Ru_x_Ce_2‐x_O_7_ (x = 0, 0.1, 0.2, 0.4) solid solutions produces catalysts with outstanding activity and stability for DRM, outperforming benchmark catalysts prepared by wetness impregnation or sodium borohydride reduction [10].

Beyond offering superior stability compared with traditional supported catalysts, exsolution‐derived catalysts can integrate multiple active sites in close proximity (e.g., exsolved NPs and active sites within the bulk reservoir), thereby facilitating intermediate transport during catalytic reactions. In this context, high‐entropy oxides (HEOs) represent a particularly promising platform for constructing highly efficient and stable catalysts via exsolution [17, 18]. HEOs are a class of oxide systems composed of multiple equimolar metal elements with tunable redox activity, variable compositions. Reductive environments (e.g., H_2_, CH_4_) can induce the formation of multicomponent alloys via exsolution from HEOs [14, 19, 20, 21]. At the atomic level, exsolution involves two fundamental steps: the breaking of metal–oxygen (M–O) bonds in the host oxide lattice and the subsequent formation of metal–metal (M–M) bonds during NPs formation [22, 23, 24]. Therefore, by precisely controlling the local environment surrounding metal atoms through controlled lattice distortion and composition variation, under reduction conditions, it is possible to tune the kinetics of M–O bond cleavage and M–M bond formation, thereby achieving fine control over exsolved NPs composition and dispersion. This, in turn, can modulate the adsorption and desorption behavior of reactants and intermediates, ultimately allowing precise control over catalytic performance. The multicomponent nature and high configurational entropy of HEOs offer a unique opportunity to tailor the local environment of metal atoms, enabling selective exsolution of specific metals and fine‐tuning of NPs characteristics and metal–support interactions. However, strategies capable of controlling the exsolution dynamics in HEOs have rarely been explored, and the fundamental factors governing exsolution in these systems remain poorly understood.

**FIGURE 1:**
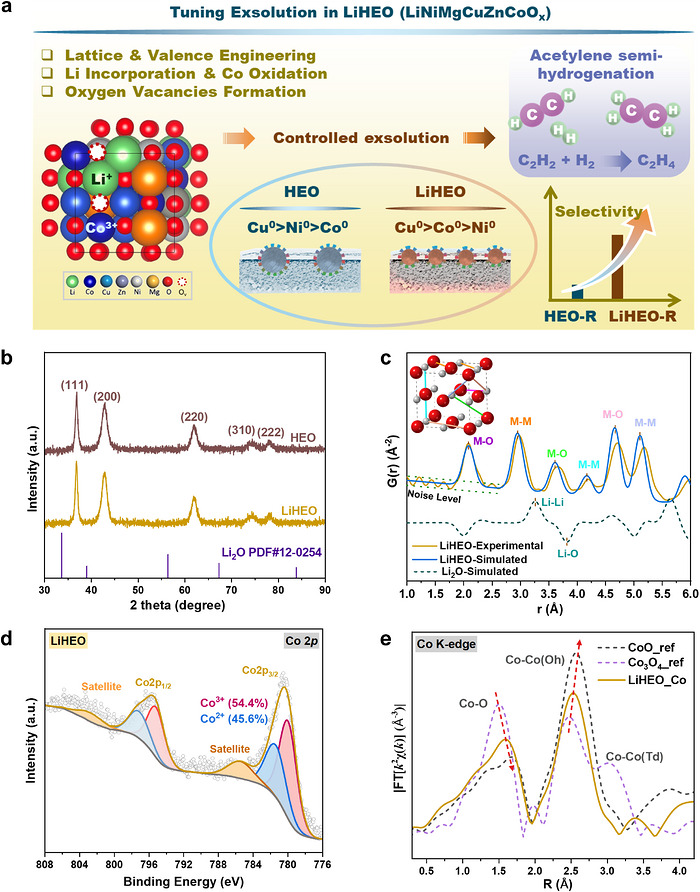
(a) Schematic illustration in tuning the exsolution behavior of HEO via lattice and valence engineering toward enhanced acetylene semi‐hydrogenation. (b) XRD patterns of HEO and LiHEO sample. (c) Experimental nPDF of LiHEO, together with the simulated patterns for LiHEO and Li_2_O. (d) Co 2p XPS spectra of LiHEO. (e) The EXAFS spectra of LiHEO at Co K‐edge with CoO and Co_3_O_4_ as references.

In this work, exsolution dynamics in HEOs are precisely tuned through a combined lattice‐ and valence‐engineering strategy, leading to a highly active and selective catalyst for acetylene semi‐hydrogenation. Incorporation of Li^+^ into the lattice of a rock salt‐structured HEO (LiNiMgCuZnCoO_x_, denoted as LiHEO) enables controlled metal exsolution by inducing local lattice distortion, generating abundant oxygen vacancies, and partially oxidizing cobalt sites (Co^2+^ → Co^3+^), thereby redistributing local charge while preserving structural integrity. This approach promotes the exsolution of catalytically active metallic Cu NPs under mild conditions and alters the exsolved nanoparticle composition from Cu^0^> Ni^0^> Co^0^ in the reduced HEO (HEO‐R) to Cu^0^> Co^0^> Ni^0^ in the reduced LiHEO (LiHEO‐R). The resulting modulation of exsolution behavior and Li‐induced lattice distortion governs hydrogen activation pathways at the catalyst surface, ultimately delivering superior activity and selectivity in acetylene semi‐hydrogenation, outperforming state‐of‐the‐art Cu‐based transition‐metal catalysts. The approach developed in this work demonstrates how entropy‐enabled lattice and valence engineering can be leveraged to control exsolution kinetics and surface chemistry in complex oxides, transforming HEOs from passive supports into programmable catalyst precursors (Figure 1a).

## 2 Results and Discussion

### 2.1 Design Principle and Local Charge Redistribution in HEOs

Exsolution behavior is commonly regulated by tuning external parameters, such as reduction temperature, duration, and gas composition, or by modifying intrinsic oxide properties to control nanoparticle size and dispersion [15, 16]. At a fundamental level, the exsolution sequence is dictated by the reduction potentials of the constituent metal species in the oxide precursor. For example, Ni_x_Mg_1‐x_O solid solutions enable the exsolution of highly dispersed and stabilized Ni NPs for CO_2_ hydrogenation, while Mg (Mg^2+^→Mg^0^, ‐2.37 eV) retains its oxidized state during reaction owing to its much lower (more negative) reduction potential compared with Ni (Ni^2+^→Ni^0^, ‐0.25 eV) [25]. For the classic rock‐salt–type HEO composed of Ni, Mg, Cu, Zn, and Co, the exsolution sequence is expected to follow the relative reduction potentials of the constituent metal species: Cu > Ni∼Co >Zn > Mg (Table S1) [26, 27]. These considerations indicate that subtle modifications to the oxide lattice by tuning the local electronic environment and oxidation states of specific metal cation, can be leveraged to direct the exsolution pathway and precisely control both the sequence and extent of metal nanoparticle formation. Therefore, in this study, we implement a lattice‐ and valence‐engineering strategy to tailor the exsolution dynamics in HEOs. Specifically, Li^+^ cation was doped into the lattice of pristine HEO (NiMgCuZnCoO_x_) assisted by mechanochemistry to tune the valence of Co species (partially oxidized from Co^2+^ to Co^3+^) in the resulting catalyst (denoted as LiHEO) and then tailor the exsolution behavior.

A classic rock salt‐type high entropy oxide (HEO) composed of Ni, Mg, Cu, Zn, and Co was used as the precursor. Li^+^ was incorporated into the HEO lattice via a mechanochemical route in an equimolar proportion relative to the other five metal elements (Figure S1), yielding LiHEO sample. The lattice structure variation and existing form of Li^+^ in LiHEO was first elucidated via X‐ray diffraction (XRD) and neutron pair distribution function (nPDF). XRD analysis of LiHEO (Figure 1b) confirmed the preservation of the rock salt structure observed in pristine HEO, while no diffraction peaks corresponding to Li_2_O (Powder Diffraction File (PDF) #12‐0254) were detected. Energy‐dispersive spectroscopy (EDS) mapping images show that Ni, Mg, Cu, Zn, and Co are uniformly distributed within the LiHEO matrix, indicating that Li incorporation did not disrupt the elemental homogeneity (Figure S2). The nPDF of LiHEO exhibits alternating M–O and O–O distances in the low‐*r* region, characteristic of a rock‐salt–type local structure (Figure 1c). Comparatively, Li_2_O displays characteristic nPDF features, including a positive Li–Li correlation at ∼3.2 Å and a negative Li–O correlation at ∼3.7 Å, neither of which are observed for LiHEO. The absence of these Li_2_O‐related signatures suggests that Li is incorporated into the HEO lattice rather than forming a separate Li_2_O phase.

The pristine HEO adopts a rock salt structure in which all constituent metal cations are in the +2 oxidation state, providing a robust and uniform lattice framework [28, 29]. The well preservation of the rock‐salt architecture upon Li^+^ doping is confirmed by both XRD and nPDF analyses, suggesting that Li^+^ is accommodated within the rock‐salt lattice without altering the long‐range crystallographic order. However, incorporation of monovalent Li^+^ into a divalent cation sublattice is expected to induce local charge perturbations. Given the intrinsic redox flexibility of transition metal cations in HEOs, one plausible compensation mechanism involves partial oxidation of redox‐active species such as Ni and Co from the +2 to higher oxidation states and defects formation, thereby contributing to charge compensation and lattice stabilization. These potential charge compensation mechanisms and associated electronic structure changes are then explored in detail using X‐ray photoelectron spectroscopy (XPS) and X‐ray absorption spectroscopy (XAS). In LiHEO, the Ni 2p, Mg 2p, Cu 2p, and Zn 2p XPS spectra all show binding energies consistent with the +2 oxidation state, indicating that these metals remain unchanged upon Li^+^ doping (Figures S3 and S4). Notably, the Co 2p XPS spectrum displays two main spin‐orbit doublets corresponding to Co 2p_3/2_ and Co 2p_1/2_, along with the characteristic satellite peaks. In addition, an extra component corresponding to Co^3+^ is clearly observed, with quantitative fitting indicating the presence of 45.6% Co^2+^ and 54.4% Co^3+^(Figure 1d), whereas in the HEO sample, Co predominantly exists as Co^2+^ (Figure S5). The O 1s XPS spectrum (Figure S6) of LiHEO exhibited a relatively weak peak at 529.5 eV from the lattice oxygen, along with pronounced contributions at 531.5 and 532.3 eV compared to undoped HEO sample, corresponding to oxygen vacancies ‐associated lattice oxygen and surface‐adsorbed oxygen species, respectively [30]. The concurrent emergence of Co oxidation and oxygen‐vacancy formation can be rationalized as a cooperative charge‐compensation response to Li^+^ incorporation.

The Co K‐edge X‐ray absorption near edge structure (XANES) spectrum of LiHEO was analyzed using CoO and Co_3_O_4_ as reference compounds (Figure S7). The absorption edge of LiHEO lies closer to that of CoO, indicating that the bulk Co remains predominantly in the Co^2+^ state, while the enhanced white‐line intensity indicates a slight perturbation of the Co–O coordination environment. The local coordination environment of Co was elucidated via extended X‐ray absorption fine structure (EXAFS) spectra at Co K‐edge (Figure 1e). The first‐shell Co‐O at ∼1.6 Å is located between those of CoO and Co_3_O_4_ reference, indicating that Co predominantly occupies octahedral sites with a partial mixed valence state between Co^2+^ and Co^3+^. The second‐shell Co–Co contributions, corresponding to octahedral sites (Co‐Co (Oh), ∼2.5 Å), also fall between CoO and Co_3_O_4_, whereas no significant tetrahedral Co–Co (Co‐Co(Td), ∼3.0 Å) is observed. These observations suggest that Co mainly resides in octahedral environments, with a minor contribution of Co^3+^ coexisting with Co^2+^ within the octahedral sites. Compared with CoO, the reduced Co–O amplitude and modified Co–Co contributions indicate partial oxidation of Co^2+^ to Co^3+^ accompanied by oxygen vacancies [31]. Ni K‐edge and Cu K‐edge XANES spectra indicate that both metals remain in the +2 oxidation state, consistent with NiO and CuO references. EXAFS analysis shows that the Ni–O (∼1.5 Å) and Cu–O (∼1.4 Å) distances match those of the respective oxides, confirming octahedral coordination and preservation of the rock salt lattice upon Li^+^ incorporation (Figure S8). Overall, these results are consistent with XPS and XANES analyses, confirming the charge compensation mechanism, whereby Li^+^ incorporation redistributes Co valence states while preserving the overall rock salt framework. The incorporation of Li^+^ perturbs the local charge balance and induces oxygen vacancies, thereby tailoring the local environment around transition‐metal cations.

### 2.2 Modulating the Exsolution Behavior in HEOs

Based on the local structural changes induced by Li^+^ doping, the influence of these electronic modifications on reduction behavior and subsequent metal exsolution under reducing conditions was further examined. The effect of Li^+^ introduction on the reducibility of HEO was first investigated by hydrogen temperature programmed reduction (H_2_‐TPR) (Figure S9). The first reduction peak of both pristine HEO and LiHEO appears at around 250°C. At higher temperatures (above 400°C), LiHEO exhibited more obvious H_2_ consumption, indicating that Li doping enhances the reducibility of M–O sites (Cu‐O, Ni‐O, and Co‐O) and promotes more extensive metal exsolution under identical conditions. The exsolution behavior of pristine HEO and LiHEO was then evaluated by in situ high temperature X‐ray diffraction (HTXRD) and near‐ambient pressure X‐ray photoelectron spectroscopy (NAP‐XPS). The in situ HTXRD under a 2%H_2_/N_2_ atmosphere revealed that no new diffraction peaks appeared for both samples below 300 °C (Figure S10). Upon heating to 400 °C, a broad diffraction feature gradually appeared in LiHEO between 42.8° and 44.4°, indicative of the formation of metallic phases. This feature overlaps with the characteristic peaks of metallic Cu^0^ (PDF#04‐0836), Ni^0^ (PDF#04‐0850), and Co^0^ (PDF#15‐0806), suggesting the concurrent exsolution of these metals. In comparison, the diffraction feature associated with metal NPs exsolution in pristine HEO appeared at a higher temperature (500 °C) and was less pronounced than in LiHEO. This observation, together with the high‐temperature features in the H_2_‐TPR profile, indicates that the introduction of Li^+^ has a more pronounced effect on the reduction and exsolution behavior of metal compositions with less negative reduction potentials, such as CuO, CoO, and NiO, and Li^+^ doping could facilitate the exsolution of transition metal NPs under identical conditions.

**FIGURE 2:**
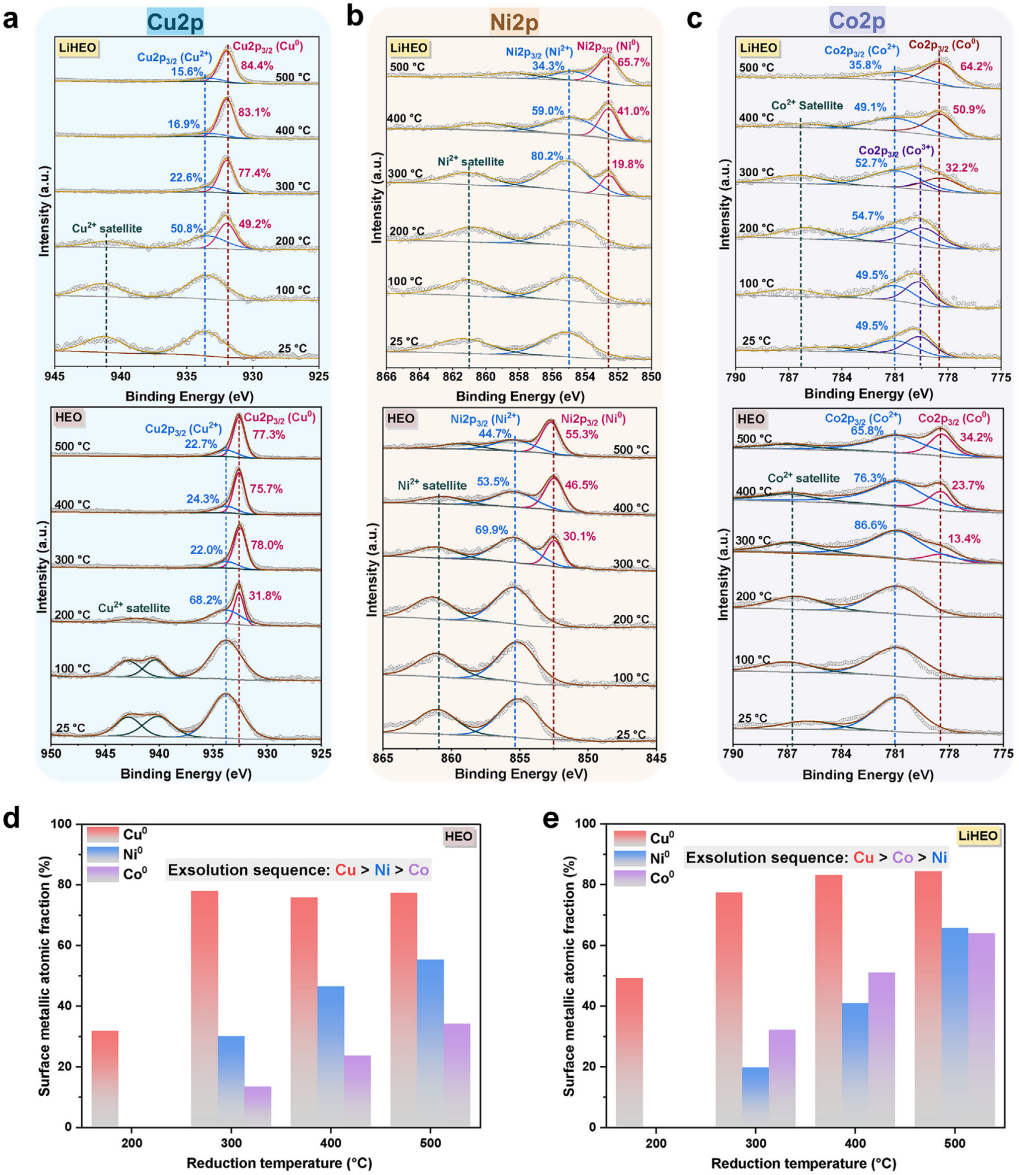
(a, b, c) NAP‐XPS spectra of LiHEO and HEO for the Cu 2p, Ni 2p, and Co 2p regions collected from 25 to 500°C under 1 mbar H_2_ atmosphere. The percentages represent the relative atomic fractions derived from the fitted peak areas. (d, e) The relative surface fractions of exsolved metallic Cu^0^, Ni^0^, and Co° for HEO and LiHEO after reduction derived from NAP‐XPS spectra.

NAP‐XPS analysis of HEO and LiHEO under 1 mbar H_2_ atmosphere revealed the progressive reduction of metal species with increasing temperature (Figure 2a,b,c). For LiHEO sample, in the Cu 2p spectra, metallic Cu (Cu^0^) was observed as early as 200°C, and its relative fraction gradually increased from 49.2% to 84.4% as the temperature rose from 200 to 500°C, accompanied by a progressive weakening of Cu^2+^ peaks (Table S2). This reduction behavior is further corroborated by the Cu LMM Auger spectra for both HEO and LiHEO (Figure S11). Specifically, the Cu LMM binding energy feature characteristic of Cu^0^ becomes increasingly pronounced with increasing reduction temperature, while the Cu^2+^‐related component correspondingly diminishes, providing independent confirmation of metallic Cu formation at elevated temperatures. In the Ni 2p spectra, metallic Ni (Ni^0^) emerged at 300 °C, with its fraction gradually increasing from 19.8% to 65.7% over the 300 – 500°C range. The Co 2p_3/2_ core level spectra could be deconvoluted into Co^3+^ and Co^2+^ species below 300°C. Upon heating to 300°C, metallic Co (Co^0^) emerged, while the fraction of Co^3+^ decreased and eventually disappeared at 400°C. By contrast, the Mg 2p and Zn 2p spectra consistently exhibited +2 oxidation states without noticeable changes throughout the entire temperature range (Figure S12). Additionally, Li 1s XPS spectrum exhibits a signal characteristic of Li^+^ in an oxide environment. Further analysis of the NAP‐XPS spectra for pristine HEO revealed that the onset temperatures for the reduction of each metal were similar to those observed in LiHEO (Table S3). Interestingly, NAP‐XPS‐derived surface metallic fractions reveal distinct exsolution sequences for HEO and LiHEO upon reduction (Figure 2d,e). Here, the exsolution sequence is defined based on the relative surface metallic fractions obtained from NAP‐XPS under identical reduction conditions and primarily reflects the kinetic preference of metal exsolution rather than a thermodynamic equilibrium trend. Both samples exhibit a strong tendency for preferential Cu° formation and exsolution, highlighting Cu as the most readily reducible and surface‐enriched species in these HEOs. In contrast, the relative exsolution behavior of Ni and Co differs between the two materials. Notably, at 400°C, pristine HEO shows surface metallic fractions of approximately Ni^0^ (46.5%), and Co^0^ (23.7%), corresponding to an exsolution sequence of Cu^0^> Ni^0^> Co^0^. Remarkably, LiHEO displays an enhanced Co° fraction (50.9%) relative to Ni^0^ (41.0%), thereby reversing the Ni and Co exsolution order to Cu^0^> Co^0^> Ni^0^. This inversion arises from Li^+^ incorporation induced valence redistribution that enriches Co^3+^ species and oxygen vacancies. Under reducing conditions, reduction of Co^3+^ necessarily proceeds via Co^2+^ as an intermediate; however, the presence of Co^3+^ substantially modifies the local electronic and coordination environment. Co^3+^ acts as an efficient electron acceptor during lattice‐oxygen removal, promoting oxygen‐vacancy formation and enhancing local defect generation. The initial Co^3+^ → Co^2+^ reduction can be regarded as a pre‐equilibrium process that induces partial relaxation and distortion of the surrounding Co–O framework. In combination with the locally oxygen‐deficient environment, this electronic and structural preconditioning weakens Co–O bonding and enhances defect‐assisted charge transport through the Co^2+^/Co^3+^ redox couple. The resulting increase in vacancy concentration and accelerated local electron redistribution lowers the local oxygen chemical potential and facilitates reduction‐driven cation migration. Consequently, Co^2+^ species associated with Li^+^‐induced defects are preferentially reduced and exsolved as metallic Co, kinetically favoring Co^0^ nucleation over Ni during the early stages of exsolution [32, 33].

Additionally, density functional theory (DFT) calculations were performed to quantify the oxygen vacancy formation energies at different metal‐centered sites in HEO and LiHEO (Figure S13). For Cu‐centered oxygen vacancies, the averaged formation energy decreases markedly from 2.86 eV in pristine HEO to 1.87 eV in LiHEO, indicating that Li incorporation facilitates oxygen removal and oxygen vacancy formation. Consistently, Li doping also lowers the vacancy formation energy at Ni sites from 3.16 to 2.95 eV. More interestingly, a more pronounced reduction is observed for Co‐associated oxygen vacancies, with the formation energy sharply reduced from 3.68 to 2.01 eV, revealing a strong Li+‐induced activation of the local Co–O coordination environment [34]. This pronounced energy changes can be attributed to the altered electronic structure induced by Li+ incorporation. As monovalent Li+ substitutes for divalent metal cations in the HEOs lattice, charge compensation is required, which weakens the local metal‐oxygen bonding and thereby lowers the energy cost for oxygen vacancies formation, which is consistent with the modified exsolution behavior observed experimentally. Based on the combined experimental observations and theoretical analysis, these results establish a direct mechanistic link between Li^+^ incorporation, Co valence evolution, oxygen vacancy formation, and the alteration in the exsolution sequence. Specifically, Li^+^‐induced valence redistribution and defect formation enhance the relative reducibility and exsolution propensity of Co compared to Ni, leading to the observed inversion in exsolution sequence.

### 2.3 Enhanced Catalytic Activity and Selectivity in Acetylene Semihydrogenation

Selective semi‐hydrogenation of acetylene to ethylene is a critical process for producing polymer‐grade ethylene. The key challenge is to maximize ethylene selectivity while minimizing over‐hydrogenation to ethane and suppressing acetylene oligomerization leading to C_4+_ hydrocarbons (green oil). [35, 36] Noble metal catalysts, particularly Pd‐based systems, exhibit high activity and selectivity but are hindered by their high cost and susceptibility to deactivation via sintering and coke formation [37, 38, 39]. Cu‐based catalysts have attracted considerable attention owing to their moderate hydrogenation activity, intrinsic selectivity for semi‐hydrogenation, and improved resistance to coking. However, their relatively low intrinsic activity often requires elevated reaction temperatures, which can compromise selectivity. [40, 41, 42, 43] Building on the capability to precisely control exsolution in HEOs, such catalysts with well‐dispersed, stable metal NPs and strong meta support interactions offer promising opportunities for selective hydrogenation of acetylene‐to‐ethylene.

**FIGURE 3:**
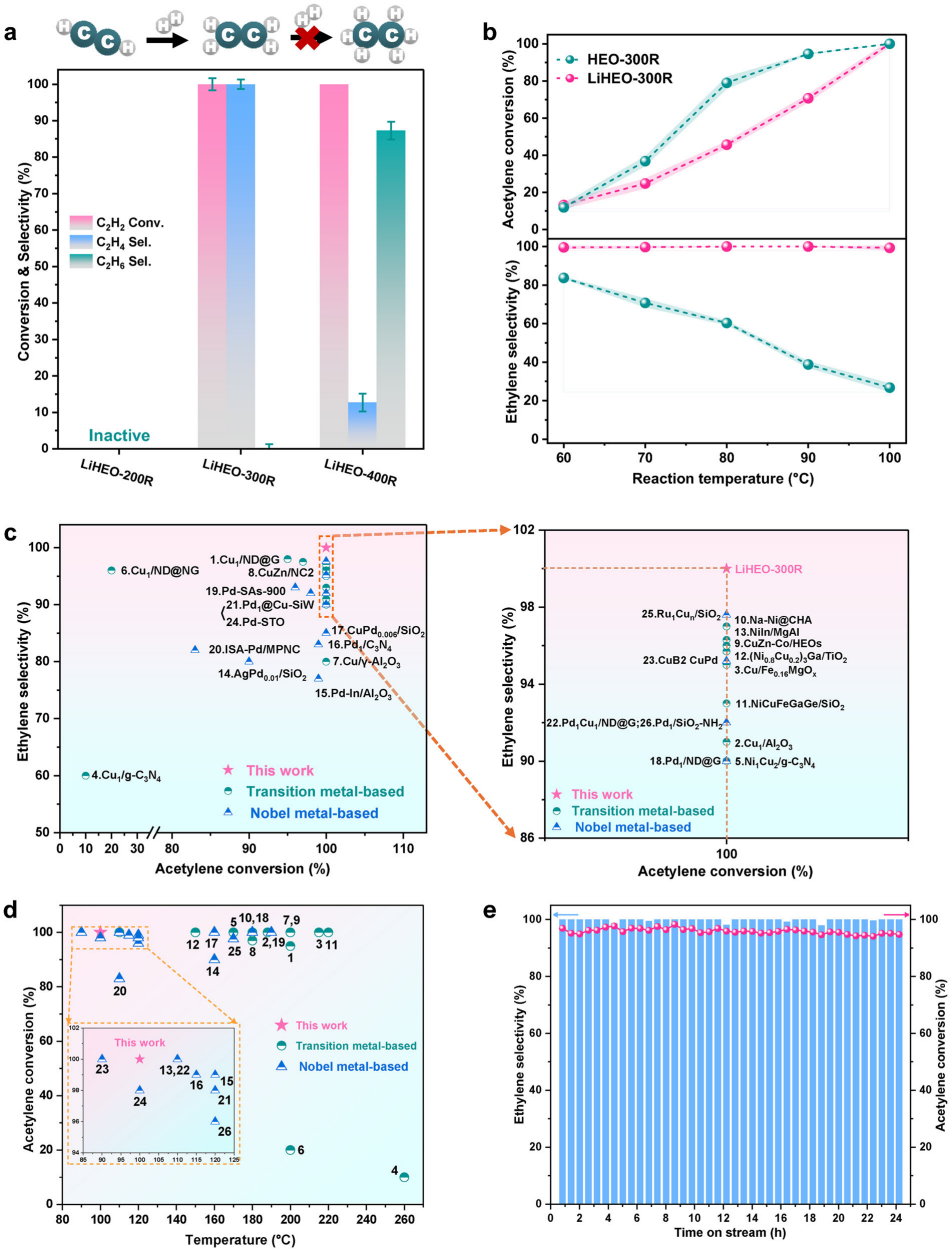
(a) Acetylene semi‐hydrogenation performance of LiHEO catalysts reduced at different temperatures (LiHEO‐200R, LiHEO‐300R, and LiHEO‐400R). Reaction conditions: 1 atm, 100°C, 2.5%H_2_/0.5%C_2_H_2_/Ar, 15000 mL·g^−1^·h^−1^. (b) Temperature‐dependent acetylene conversion and ethylene selectivity over HEO‐300R and LiHEO‐300R (1 atm, 2.5%H_2_/0.5%C_2_H_2_/Ar, 15000 mL·g^−1^·h^−1^). Error bars represent the standard deviation from repeated catalytic measurements (n = 3). (c) Diagram of acetylene conversion and ethylene selectivity of different acetylene hydrogenation catalysts (refs. listed in **Table** **S4**). (d) Comparison of acetylene conversion as a function of reaction temperature for the current and literature‐reported catalyst. Numbers in the figure denote the catalysts as indexed in Table S4. (e) Stability test of LiHEO‐300R (1 atm, 2.5%H_2_/0.5%C_2_H_2_/Ar, 15000 mL·g^−1^·h^−1^).

The as‐synthesized HEO catalysts were subsequently evaluated for acetylene semi‐hydrogenation after reducing pretreatment. LiHEO catalysts pre‐reduced at 200, 300, and 400°C under 5% H_2_/Ar atmosphere are denoted as LiHEO‐200R, LiHEO‐300R, and LiHEO‐400R, respectively, and used for acetylene hydrogenation. Under identical reaction conditions, their catalytic performance shows a pronounced dependence on the reduction temperature (Figure 3a). LiHEO‐200R exhibits negligible activity, whereas LiHEO‐300R delivers nearly complete acetylene (C_2_H_2_) conversion accompanied by ∼100% ethylene (C_2_H_4_) selectivity at reaction temperature of 100°C. LiHEO‐400R also attains full conversion but suffers a drastic decline in C_2_H_4_ selectivity to 12.7%. These results demonstrate that higher reduction temperatures enhance reactivity but severely compromise selectivity, with LiHEO‐300R providing the most favorable balance between C_2_H_2_ conversion and C_2_H_4_ selectivity. Motivated by the outstanding performance of LiHEO‐300R, we benchmarked it against HEO‐300R to assess the extent of improvement achieved through Li modification. As shown in Figure 3b, both catalysts exhibit similar reaction temperature‐dependent conversion trends; however, LiHEO‐300R consistently maintains near 100% ethylene selectivity across the entire temperature range (60–100°C). In contrast, HEO‐300R undergoes pronounced over‐hydrogenation, with the selectivity declining dramatically to 26.7% at 100°C. Remarkably, LiHEO‐300R achieves complete acetylene conversion while preserving 100% selectivity at 100°C, underscoring its superior selective hydrogenation capability. To enable a direct comparison with previously reported catalysts, an acetylene conversion‐ethylene selectivity plot was employed (Figure 3c and Table S4), which clearly distinguishes catalysts capable of maintaining high selectivity at elevated conversion. LiHEO‐300R resides at the optimal corner of this plot, achieving full C_2_H_2_ conversion with 100% C_2_H_4_ selectivity, thereby surpassing state‐of‐the‐art noble‐metal and transition‐metal systems. Because achieving complete conversion at low temperatures remains particularly challenging for transition metal catalysts, reaction temperature was further used as a metric (Figure 3d and Table S4). LiHEO‐300R reaches 100% conversion at only 100°C, markedly lower than temperatures required for previously reported catalysts, including majority of noble‐metal benchmarks, while simultaneously delivering reaction rates that surpass most reported transition‐metal catalysts. Moreover, LiHEO‐300R demonstrates excellent durability, sustaining nearly constant acetylene conversion around 95% and 95%–98% ethylene selectivity over 24 h at 100°C (Figure 3e). For comparison, a Cu/MgO–ZnO reference catalyst shows negligible activity under identical conditions despite the formation of metallic Cu (Figure S14), highlighting the important role of the HEO matrix in modulating the catalytic behavior of Cu^0^ species. Collectively, these results underscore the exceptional catalytic efficiency and promising potential of LiHEO‐300R for selective acetylene semi‐hydrogenation.

### 2.4 Reaction Pathway and Structure‐Performance Elucidation

To uncover the structural origins underlying the observed performance differences, detailed comparative characterizations of the reduced LiHEO and HEO catalysts after exsolution were performed. Inductively coupled plasma (ICP) analysis shows that the elemental compositions of the catalysts remain unchanged after reduction (Figure S15). High‐angle annular dark‐field scanning transmission electron microscopy equipped with energy‐dispersive X‐ray spectroscopy (HAADF‐STEM EDS) mapping confirms that Cu shows localized enrichment in certain regions for both HEO‐300R and LiHEO‐300R (Figures S16 and S17); however, the overall elemental distribution remains uniform, with all elements, including Cu, preserving their dispersion across the high‐entropy matrix.

**FIGURE 4:**
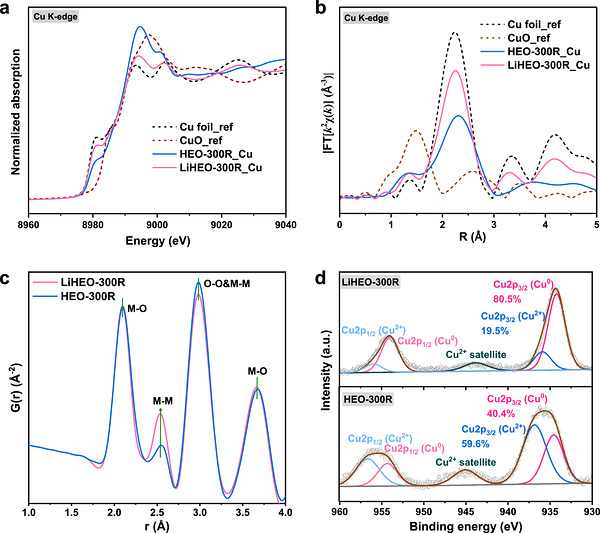
(a) XANES and (b) EXAFS spectra collected at Cu K‐edge; (c) Neutron PDF results; (d) XPS profiles collected at Cu 2p regions after Ar sputtering for HEO‐300R and LiHEO‐300R.

XANES and EXAFS analyses were further conducted to probe the electronic states and local environments of Cu, Ni, and Co sites. The Cu K‐edge XANES spectra of HEO‐300R and LiHEO‐300R are presented in Figure 4a. HEO‐300R exhibits an absorption edge slightly above that of Cu foil, with an intermediate white line intensity, indicating the coexistence of metallic Cu^0^ and residual Cu^2+^. In contrast, LiHEO‐300R shows a pronounced near‐edge feature around 8980 eV and a markedly weaker white line, demonstrating that Li doping significantly promotes the reduction of Cu species, which is consistent with the in situ HTXRD and NAP XPS results. Complementary Cu K‐edge EXAFS analysis further supports this observation (Figure 4b). In both HEO‐300R and LiHEO‐300R, the Cu‐O scattering at ∼1.9 Å persists, suggesting the presence of residual Cu^2+^. Significant differences are observed in the Cu–Cu scattering at ∼2.3 Å, with LiHEO‐300R exhibiting a much stronger peak, consistent with enhanced Cu reduction [41]. In contrast to Cu, both Ni and Co remain fully oxidized after reduction. As demonstrated by the respective K‐edge XANES spectra (Figure S18), no features characteristic of metallic states is observed. Consistently, the EXAFS spectra display only metal–oxygen scattering contributions (Ni–O and Co–O at ∼1.5 Å), with no detectable metal–metal contributions (Ni–Ni ∼2.1 Å; Co–Co ∼2.2 Å), confirming that neither Ni nor Co undergoes reduction under the applied pretreatment conditions at 300°C. Correspondingly, their EXAFS spectra exhibit only metal–oxygen scattering (Ni–O and Co–O at ∼1.5 Å) and no metal–metal contributions (Ni–Ni ∼2.1 Å; Co–Co ∼2.2 Å), confirming the absence of metallic Ni^0^ or Co° formation [44, 45].

From the nPDF patterns, both reduced samples exbibit, relative to the fresh sample (Figure 1c), a distinct feature at ∼2.5 Å, which can be assigned to short‐range metal‐metal (M‐M) correlations. Notably, this M–M peak is substantially intensified in LiHEO‐300R compared to HEO‐300R, indicating that Li incorporation greatly promotes the formation of short‐range metallic contacts (Figure 4c).

XPS was also employed to investigate the surface chemical states in HEO‐300R and LiHEO‐300R, and the spectra were collected after Ar sputtering to probe the near‐surface lattice environment. The Cu 2p spectra of HEO‐300R and LiHEO‐300R display characteristic signals of both Cu^2+^ and metallic Cu (Cu^0^), consistent with the Cu exsolution inferred from XAS analyses. Based on peak‐fitting analysis, HEO‐300R contains approximately 59.6% Cu^2+^ and 40.4% Cu^0^, whereas LiHEO‐300R exhibits a substantially higher metallic fraction (19.5% Cu^2+^ and 80.5% Cu^0^), highlighting the markedly enhanced Cu exsolution induced by Li incorporation (Figure 4d). For HEO‐300R, the Ni 2p and Co 2p spectra are dominated by signals characteristic of Ni^2+^ and Co^2+^, suggesting that Ni and Co cations remain predominantly in the oxidized state at the surface. In the case of LiHEO‐300R, Ni 2p spectra also indicates Ni^2+^, while the Co 2p spectra reveals the coexistence of Co^3+^ and Co^2+^, with Co^3+^ and Co^2+^ accounting for 21.6% and 78.4%, respectively (Figure S19). In contrast, the unreduced LiHEO sample exhibits a higher Co^3+^ fraction of 54.4%, indicating partial reduction of Co^3+^ after reduction pretreatment. The persistence of mixed‐valence Co species suggests redox adaptability within the entropy‐stabilized lattice. In addition, the Mg 1s, Mg 2p and Zn 2p spectra of both samples exhibit binding energies characteristic of Mg^2+^ and Zn^2+^, confirming that these elements remain stable in the +2 oxidation state (Figure S20). This suggests that the HEO framework is preserved, while the observed exsolution phenomena occur without disrupting the overall lattice integrity. The O 1s spectra can be deconvoluted into multiple components corresponding to lattice oxygen (O_lat_, ∼529.5 eV), oxygen associated with oxygen vacancies (O_vac_, ∼531 eV), and surface adsorbed oxygen species (O_ads_, ∼532 eV) (Figure S21). LiHEO‐300R exhibits a markedly higher O_vac_ contribution, directly indicating promoted oxygen vacancy formation induced by Li incorporation. This enhanced vacancy population is consistent with the reduced vacancy formation energies predicted by DFT and correlates with stabilized mixed Co^3+^/Co^2+^ states via charge compensation. The combined characterization results indicate that the catalytically active sites originate from exsolved metallic Cu, whose formation is strongly facilitated by Li incorporation.

Based on the observed changes in catalytic activity (Figure 3a), higher reduction temperatures are expected to influence the extent of metal exsolution and correspond to markedly enhanced activity. XPS analysis reveals a pronounced change in surface electronic states with the increasing reduction temperatures from 200 to 400°C. The LiHEO‐200R sample preserves oxidized features for all examined elements (Ni, Mg, Cu, Zn, and Co) with no detectable metallic components (Figure S22), whereas LiHEO‐400R shows distinct low‐binding‐energy contributions indicative of metallic Cu, Co, and Ni, while Mg and Zn remain divalent (Figure S23). At the Cu, Ni, and Co K‐edges, XANES spectra of LiHEO‐200R closely resemble oxidic reference, with XANES edge positions characteristic of fully oxidized species, and the EXAFS are dominated by short‐range M–O scattering with no resolvable M–M contributions. In contrast, LiHEO‐400R exhibits lower‐energy edge shifts and pronounced M–M scattering at ∼2.1 Å, confirming the reduction and exsolution of metallic Cu, Ni, and Co (Figures S24–S26). Together with these results, LiHEO‐200R does not form metallic Cu active sites, consistent with the negligible low‐temperature reducibility observed in H_2_‐TPR and its negligible catalytic activity. Reduction at 400°C generates a multi‐metallic Cu–Ni–Co phase, which leads to markedly enhanced catalytic activity but also promotes excessive hydrogenation. Therefore, the catalytic selectivity can be directly correlated with the nature of the active sites, where the absence of metallic Cu results in inactivity, the formation of moderately reduced Cu‐based sites at intermediate temperature favors selective hydrogenation, and the development of multi‐metallic Cu–Ni–Co ensembles at higher reduction temperature promotes over‐hydrogenation. By comparison, LiHEO‐300R exhibits enhanced Cu exsolution relative to HEO‐300R subjected to the same reduction treatment yet displays lower catalytic activity and higher C_2_H_4_ selectivity under low temperature reaction conditions (< 100°C). Additionally, XRD patterns of spent HEO and LiHEO show that the main crystalline structure is largely retained, similar to that observed for the sample reduced at 300°C, indicating minor structural evolution (Figure S27). XPS analysis of the spent samples further reveals that Cu remains in the metallic state, while Mg, Zn, Ni, and Co are predominantly oxidized, consistent with the mild reaction conditions (Figures S28 and S29). These results suggest that the catalyst structure and surface chemical states remain stable during the reaction. Mg and Zn remain in a divalent state across different reduction temperatures and are not directly involved as active sites but may influence the exsolution behavior through their structural role within the HEO lattice. These observations indicate that catalytic activity and selectivity are not solely dictated by Cu exsolution but are strongly influenced by Li‐dependent modulation of H_2_ dissociation and hydrogenation.

To elucidate the reaction pathway and examine hydrogen activation and migration, in situ diffuse reflectance infrared Fourier transform spectroscopy (DRIFTS) under C_2_H_2_ hydrogenation conditions, inelastic neutron scattering (INS) and H_2_–D_2_ isotopic exchange measurements were conducted to probe C_2_H_2_ adsorption and H_2_ dissociation behaviors on Li^+^‐modified catalysts. Complementary ethylene pulse hydrogenation experiments were used to assess surface hydrogen availability and reactivity under reaction‐relevant conditions, offering a mechanistic perspective on hydrogenation behavior. Both HEO and LiHEO were pretreated at 300°C for 1 h in 5%H_2_/He prior to the spectroscopic measurements. Under C_2_H_2_ hydrogenation conditions, the in situ DRIFTS spectra of LiHEO display intense features associated with gaseous acetylene (3313 and 3263 cm^−1^), which are readily removed upon He purging (Figure S30). In the fingerprint region, multiple acetylene‐derived bands appear between 1600 and 1300 cm^−1^, indicating the formation of surface intermediates during C_2_H_2_ hydrogenation. Notably, several of these features remain detectable after He purging, suggesting the presence of relatively strongly bound acetylene‐derived species. In addition, a weak band attributable to weakly adsorbed ethylene or partially hydrogenated C_2_H_x_ species (1720 cm^−1^) is observed on LiHEO [46, 47]. By comparison, pristine HEO exhibits fewer acetylene‐derived features in the fingerprint region, which are largely removed upon He purging, indicating weaker acetylene adsorption. The contrast in the intensity and persistence of acetylene‐derived species highlights a clear difference in adsorption strength between reduced pristine HEO and LiHEO.

**FIGURE 5:**
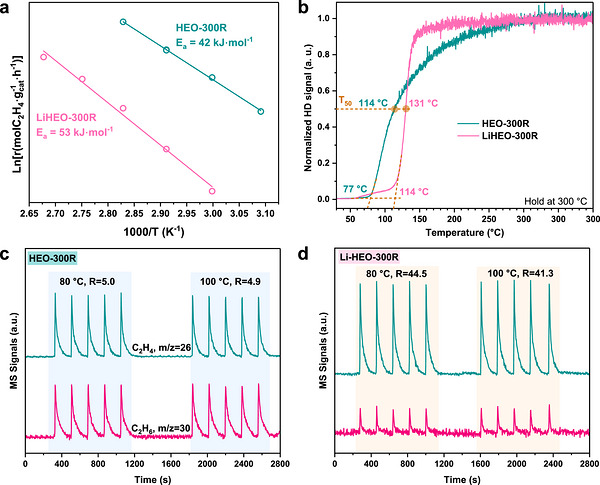
(a) Arrhenius plots for acetylene semi‐hydrogenation, showing apparent activation energies (E_a_). (b) Normalized HD formation profiles as a function of temperature. (c, d) Ethylene pulse hydrogenation at 80 °C and 100 °C monitored by mass spectrometry, R denotes the average ratio of ethylene (m/z = 26) to ethane (m/z = 30) signal areas.

INS analysis shows that pristine LiHEO contains Li‐associated hydroxyls, being a mixture of LiOH‐H_2_O and LiOH, LiOH persists after reduction pretreatment (LiHEO‐300R) (Figure S31) [48], together with the larger H_2_ consumption observed in H_2_‐TPR (Figure S9), suggest that Li incorporation may modify the H_2_ dissociation pathway and hydrogenation capabilities of HEO catalyst. This inference is further supported by apparent activation‐energy measurements for acetylene semi‐hydrogenation, where LiHEO‐300R exhibits a higher apparent activation energy (53 kJ·mol^−1^) than that of HEO‐300R (42 kJ·mol^−1^), indicating suppressed hydrogenation kinetics (Figure 5a). The normalized H_2_–D_2_ exchange profiles (Figure 5b) reveal distinct hydrogen activation behaviors for two samples (LiHEO‐300R and HEO‐300R) that correlate with catalytic performance. In HEO‐300R, HD formation starts at low temperature and increases gradually (77°C) and reaches half‐conversion (T_50_) at 114°C, consistent with higher low‐temperature activity (<100 °C) but pronounced over‐hydrogenation. By contrast, LiHEO‐300R exhibits a sharp HD increase over a narrow temperature range, with the rapid‐growth temperature at ∼114 °C and T_50_ = 131 °C, corresponding to complete conversion around 100 °C with almost 100% ethylene selectivity [49]. Structural characterization shows that Li incorporation promotes Cu exsolution, which alters the local electronic and geometric environment of surface sites and provides a mechanistic basis for both the modified H_2_ dissociation and the optimized selectivity. These observations indicate that the differences in HD profiles reflect variations in site distribution and activation behavior rather than total site number, linking surface structure directly to hydrogenation performance. Ethylene pulse hydrogenation was conducted at 80 and 100 °C to further probe hydrogenation behavior, with product distribution monitored by mass spectrometry (Figure 5c,d). The ratio of ethylene (m/z = 26) to ethane (m/z = 30) signal areas (R  =  C_2_H_4_/C_2_H_6_) was used as a measure of hydrogenation ability, where a higher R denotes suppressed ethylene hydrogenation and a lower R indicates enhanced hydrogenation activity. At both temperatures, HEO‐300R shows lower R values (5.0 for 80°C, 4.9 for 100°C) than LiHEO‐300R, indicating a stronger hydrogenation activity and a tendency toward over‐hydrogenation. In contrast, LiHEO‐300R exhibits significantly much higher R values (44.5 for 80°C, 41.3 for 100°C) under identical conditions, consistent with a weakened hydrogenation ability [50, 51]. These results align with the H_2_–D_2_ exchange profiles, where Li doping delayed hydrogen activation, suggesting that Li incorporation modifies surface Cu sites that suppresses excessive hydrogenation while maintaining overall conversion efficiency.

DFT calculations were performed to examine the acetylene hydrogenation pathway on HEO‐300R and LiHEO‐300R (Figure S32). After formation of the *C_2_H_4_ intermediate, ethylene can either desorb or undergo further hydrogenation to *C_2_H_5_ + *H. On LiHEO‐300R, the barrier for further hydrogenation is higher than the desorption energy, favoring *C_2_H_4_ desorption. In contrast, on HEO‐300R, further hydrogenation is kinetically preferred and leads to a strongly stabilized *C_2_H_5_ + *H intermediate, promoting over‐hydrogenation. These results indicate that selectivity is governed by the competition between ethylene desorption and further hydrogenation, rather than adsorption strength alone, consistent with the enhanced ethylene selectivity observed on LiHEO‐300R.

## 3 Conclusion

This work demonstrates that precise modulation of lattice and valence in HEOs provides a facile and powerful tool to control metal exsolution dynamics and surface reactivity. Li^+^ incorporation induces local lattice distortion, generates oxygen vacancies, and partially oxidizes Co, collectively steering the sequential exsolution of Cu, Co, and Ni NPs and stabilizing their dispersion. These structural and electronic modifications directly regulate surface hydrogen activation pathways, enabling nearly quantitative acetylene conversion with exceptional ethylene selectivity at low reaction temperatures. This study establishes that entropy‐stabilized multicomponent oxides can serve as programmable catalyst scaffolds, and rational tuning of local coordination and redox environments allows predictive control over exsolved nanoparticle composition and catalytic performance. The insights provide a generalizable strategy for designing next‐generation heterogeneous catalysts with tailored activity, selectivity, and stability through subtle structure engineering.

## Conflicts of Interest

The authors declare no conflicts of interest.

## Supporting information




**Supporting file**: anie72708‐sup‐0001‐SuppMat.docx.

## Data Availability

The data that support the findings of this study are available from the corresponding author upon reasonable request.
