# Supplementary material for: Controlling Exsolution Dynamics in High‐Entropy Oxides for Highly Active and Selective Acetylene Semi‐Hydrogenation
Source: Angew Chem Int Ed Engl. 2026 May 15;65(28):e9920205. doi: 10.1002/anie.9920205 (PMC13340525; doi:10.1002/anie.9920205)
Supplement: Supplementary file 1 — Supporting file: anie72708‐sup‐0001‐SuppMat.docx. [file ANIE-65-e9920205-s001.docx]

Controlling Exsolution Dynamics in High-Entropy Oxides for Highly Active and Selective Acetylene Semi-hydrogenation

Hailing Yu,^[a], [b], †^ Caiqi Wang,^[b], †^ Kevin M. Siniard,^[a]^ Qingju Wang, ^[a]^ Yuanpeng Zhang, ^[c]^ J. Anibal Boscoboinik,^[d]^ Tong Xiao,^[d]^ Eliseo Perez Gomez^[d]^ Shuai Yuan,^[e]^ Arun S. Asundi,^[f]^ Oliver Mueller,^[f]^ Murillo Longo Martins,^[c]^ Yongqiang Cheng,^[c]^ Michael Richard Koehler,^[g]^ De-en Jiang,^[e]^ Zili Wu,^[b]^ Zhenzhen Yang,*^[b]^ and Sheng Dai*^[a], [b]^

[a] Dr. H. Yu, K. M. Siniard, Dr. Q. Wang, and Dr. S. Dai
Department of Chemistry, Institute for Advanced Materials and Manufacturing
University of Tennessee, Knoxville

Knoxville, TN, 37996, USA.

[b] Dr. H. Yu, Dr. C. Wang, Dr. Z. Wu, Dr. Z. Yang, and Dr. S. Dai
Chemical Sciences Division
Oak Ridge National Laboratory
Oak Ridge, TN, 37831, USA.

Email: yangz3@ornl.gov; dais@ornl.gov

[c] Dr. Y. Zhang, Dr. M. Martins, and Dr. Y Cheng,

Neutron Scattering Division

Oak Ridge National Laboratory

Oak Ridge, TN 37831, USA.

[d] Dr. T. Xiao, Dr. J. A. Boscoboinik, and E. P. Gomez

Center for Functional Nanomaterials

Brookhaven National Laboratory

Upton, NY 11973-5000, USA.

[e] S. Yuan, and Dr. D. Jiang

Department of Electrical and Computer Engineering

Vanderbilt University

Nashville, TN 37235, USA.

[f] Dr. A. S. Asundi, and Dr. O. Mueller

Stanford Synchrotron Radiation Lightsource

SLAC National Accelerator Laboratory

Menlo Park, California 94025, USA.

[g] Dr. M. R. Koehler

Institute for Advanced Materials and Manufacturing Diffraction Facility
University of Tennessee, Knoxville

Knoxville, TN, 37996, USA.

^†^ These authors contributed equally to this work.

**Table of Contents**

Experimental Procedures 4

Catalyst synthesis 4

Characterizations 4

Acetylene semi-hydrogenation performance evolution 6

Supplementary Figures 8

Figure S1. Molar fractions of individual metals in HEO (a) and LiHEO (b) obtained from ICP measurements. 8

Figure S2. HAADF-STEM-EDS mapping results of fresh LiHEO. 9

Figure S3. Ni 2p, Mg 2p, Cu 2p, and Zn 2p XPS spectra of fresh LiHEO. 10

Figure S4. Ni 2p, Mg 2p, Cu 2p, and Zn 2p XPS spectra of fresh HEO. 11

Figure S5. Co 2p XPS spectra of fresh HEO. 12

Figure S6. O 1s XPS spectra of fresh HEO and LiHEO samples. 13

Figure S7. Co K-edge XANES spectra of the fresh LiHEO sample. 14

Figure S8. Cu and Ni K-edge XANES and EXAFS spectra of the fresh LiHEO sample. 15

Figure S9. H_2_-TPR profiles of HEO and LiHEO samples. 16

Figure S10. In situ HTXRD patterns of HEO (a) and LiHEO (b) collected from 25 to 500 °C under a 2%H_2_/N_2_ atmosphere. 17

Figure S11. NAP-XPS spectra of HEO and LiHEO for the Cu LMM regions collected from 25 to 500°C. 18

Figure S12. NAP-XPS spectra of LiHEO for the Zn 2p, Li 1s, and Mg 2p regions collected from 25 to 500°C. 19

Figure S13. The DFT model structures of (a) HEO and (b) LiHEO. (c) The average oxygen vacancy formation energy of Cu, Ni, and Co sites. 20

Figure S14. (a) XRD patterns of the Cu/MgO–ZnO reference catalyst before and after reduction in 5% H_2_/Ar at 300 °C, showing the transformation of CuO to metallic Cu.; (b) comparison of acetylene conversion over LiHEO-300R and Cu/MgO–ZnO-300R under identical conditions, highlighting the negligible activity of the reference catalyst.(1 atm, 2.5%H_2_/0.5%C_2_H_2_/Ar, 15000 mL·g^-1^·h^-1^). 21

Figure S15. Elements composition analysis results of spent HEO-300R (a) and spent LiHEO-300R (b) samples. 22

Figure S16. HAADF-STEM-EDS mapping images of LiHEO-300R sample. 23

Figure S17. HAADF-STEM-EDS mapping images of HEO-300R sample. 24

Figure S18. Ni and Co K-edge XANES and EXAFS spectra of HEO-300R and LiHEO-300R samples. 25

Figure S19. XPS spectra of the Ni 2p and Co 2p regions for HEO-300R and LiHEO-300R samples. 26

Figure S20. XPS spectra of the Zn 2p, Mg 1s for HEO-300R (a, b) and Zn 2p and Mg 2p regions for LiHEO-300R (c, d). 27

Figure S21. O 1s XPS spectra of HEO-300R (a) and LiHEO-300R (b) samples. 28

Figure S22. XPS spectra of LiHEO-200R sample. 29

Figure S23. XPS spectra of LiHEO-400R sample. 30

Figure S24. Cu K-edge XANES and EXAFS spectra of LiHEO, LiHEO-200R, LiHEO-300R, and LiHEO-400R samples. 31

Figure S25. Ni K-edge XANES and EXAFS spectra of LiHEO, LiHEO-200R, LiHEO-300R, and LiHEO-400R samples. 32

Figure S26. Co K-edge XANES and EXAFS spectra of LiHEO, LiHEO-200R, LiHEO-300R, and LiHEO-400R samples. 33

Figure S27. XRD patterns of spent HEO and LiHEO samples, showing that the main crystalline features are largely preserved, with a weak shoulder at ~44° attributed to metallic species formed during reduction. 34

Figure S28. XPS spectra of spent HEO sample, indicating that Cu remains predominantly in the metallic state, while Mg, Zn, Ni, and Co are largely retained in their oxidized states. 35

Figure S29. XPS spectra of spent LiHEO sample, indicating that Cu remains predominantly in the metallic state, while Mg, Zn, Ni, and Co are largely retained in their oxidized states. 36

Figure S30. In situ DRIFTS spectra of HEO and LiHEO collected under C_2_H_2_ hydrogenation conditions and after He purging. 37

Figure S31. INS spectra of fresh and reduced HEO and LiAl-HEO compared to reference samples. 38

Figure S32. (a) DFT models of exsolved Cu clusters on LiHEO-300R (left) and HEO-300R (right) surfaces, and (b) the corresponding reaction energy profiles for C_2_H_2_ hydrogenation. 39

Table S1 Standard reduction potential of different metal cations. 40

Table S2. Relative contents of fitted NAP-XPS spectra for Cu 2p, Ni 2p, and Co 2p for LiHEO sample. Numbers indicate peak positions (in eV), and values in parentheses represent the corresponding fractions. 41

Table S3. Relative contents of fitted NAP-XPS spectra for Cu 2p, Ni 2p, and Co 2p for HEO sample. Numbers indicate peak positions (in eV), and values in parentheses represent the corresponding fractions. 42

Table S4. Comparison of catalytic performance (e.g., conversion, selectivity) of the present catalysts with previously reported transition metal-based and noble metal-based catalysts. 43

References 44

# Experimental Procedures

## **Catalyst synthesis**

Initial oxides MgO, ZnO, CuO, CoO, NiO, Li_2_O, all from Alfa Aesar, were used as received. For the synthesis NiMgCuZnCoO_x_ (HEO), the mixture of oxides with the equal molar ratio were weighed and put into the stainless–steel milling pot and ball–milled with 4 steel balls with 10 mm diameter for 2 h at 25 Hz. The mixtures were heated in air at 900 °C for 4 h and finally quenched to room temperature to stabilize the high-entropy phase. For the Li-NiMgCuZnCoO_x_ (LiHEO) sample, 0.045 g Li_2_O were mixed with 1 g HEOs sample and then ball milling 30 minutes with the above conditions. For comparison, under the same treatment conditions, HEO was ball-milled for 30 min. Cu/MgO–ZnO reference catalyst was prepared by a conventional impregnation method. Commercial MgO and ZnO were used as supports. Copper nitrate (Cu(NO_3_)_2_ 3H_2_O) were used as metal precursors. The metal loadings were adjusted to match the metal composition ratios in the HEO sample. The desired amounts of metal precursors were dissolved in deionized water and impregnated onto the mixed oxide support, followed by drying and calcination at 500 °C for 4 h in air.

## **Characterizations**

*Inductively coupled plasma-optical emission spectroscopy (ICP-OES)* measurements were performed using an Agilent 5110 instrument equipped with an Agilent SPS 4 autosampler. A precisely weighed amount of each powdered sample was digested using aqua regia (a mixture of concentrated HCl and HNO_3_ in a 3:1 volume ratio) to ensure complete dissolution of the metal components. The resulting solution was then diluted with deionized water to a final volume suitable for analysis.

*Powder X-ray diffraction (PXRD)* measurements were carried out on a Malvern PANalytical Empyrean diffractometer operating at 45 kV and 40 mA. The diffraction data were acquired using a step interval of 0.02 º over 20 – 90 º, employing Cu Kα radiation (λ = 0.1540598 nm) as the source. In situ XRD patterns were continuously recorded in the HTK 1200N oven chamber made by Anton Paar under a diluted hydrogen atmosphere (2 vol% H_2_ in Ar) while heating the sample from 30 to 500 °C at a rate of 10 °C min^-1^.

The neutron diffraction and pair distribution function (nPDF) data were gathered at the Nanoscale-Ordered Materials Diffractometer (NOMAD) instrument located at the Spallation Neutron Source (SNS) within Oak Ridge National Laboratory (ORNL). The sample were loaded into the 3mm quartz capillaries. Four scans of 24 minutes each were acquired per sample and subsequently combined to enhance data statistics. The scattering signal from the empty quartz capillary measurement was subtracted to account for background, and the resulting data were normalized using the scattering intensity from a 6 mm vanadium rod to compensate for detector efficiencies. In the reduction of PDF data, a Qmax threshold of 30 Å^−1^ was applied to LiHEO, LiHEO-200R, LiHEO-300R and LiHEO-400R samples, during the Fourier transform process from S(Q) to the reduced PDF G(r) or pair distribution function g(r). Structure refinements were carried out in the TOPAS v7 software.

*X-ray photoelectron spectroscopy (XPS)* X-ray photoelectron spectroscopy (XPS) experiments were carried out in an ultrahigh vacuum (UHV) system with base pressures < 2 x 10^-9^ Torr, equipped with a hemispherical electron energy analyzer (SPECS, PHOIBOS 100) and twin anode X-ray source (SPECS, XR50). Al Kα (1486.7 eV) radiation was used at 15 kV and 20 mA. The angle between the analyzer and X-ray source was 55°, and photoelectrons were collected along the sample surface normally. All binding energies were referenced to the C 1s peak of adventitious carbon at 284.8 eV. Data processing and spectral fitting were performed using Avantage software.

*Near-ambient pressure XPS (NAP-XPS)* experiments were performed at the Center for Functional Materials at Brookhaven National Laboratory equipped with a lab-based APXPS system (SPECS Surface Nano Analysis). A monochromatized Al Kα X-ray source (hν = 1486.6 eV), focused to a ca. 300 μm diameter spot size and fixed at 55° from the sample normal, and a differentially pumped hemispherical analyzer were used for acquiring XPS spectra. Survey spectra were collected using a pass energy of 50 eV and a step size of 1 eV, and higher-resolution spectra were collected with a 30 eV pass energy. The samples were pressed to a cleaned thin Cu foil, transferred into the main analysis chamber and aligned at a sample to cone distance of 600 μm. Spectra were acquired at the stated conditions of H_2_ pressure and temperature. H_2_ was delivered to the chamber using a precision variable leak valve and the sample was heated using an IR laser source shining at the bottom of the sample holder. The temperature was measured using a type-K thermocouple attached to the sample. H_2_ was introduced before starting the temperature ramp. The ramping rate between temperatures was approximately 20 °C/min. The sample was kept at each temperature for approximately 50 min.

*X-ray absorption spectroscopy (XAS)*, including XANES and EXAFS, was conducted at beamline 2-2 at Stanford Synchrotron Radiation Lightsource (SSRL) for Cu, Ni, Co K-edges. Energy calibration was performed against corresponding metal foils. Samples were pressed into pellets with boron nitride, sealed in Kapton foil, and measured in transmission mode. Data processing and normalization were carried out using the *Demeter* software package^[1]^.

*High-angle annular dark-field scanning transmission electron microscopy (HAADF-STEM)* images, along with energy dispersive X-ray spectroscopy (EDS) elemental maps for fresh LiHEO, and spent HEO and LiHEO samples, were acquired using a Fisher Scientific Spectra 300 microscope operated at 200 kV.

*Hydrogen temperature-programmed reduction (H_2_-TPR)* experiments were measured on a Micromeritics AutoChem II 2920 system. 20 mg of catalyst was placed in a U-shaped quartz reactor and pretreated under a He stream at 150 °C for 2 h. After cooling to 50 °C, reduction was carried out in a 10% H_2_/Ar mixture (30 mL·min^-1^) while heating from 50 °C to 700 °C at a rate of 10 °C·min^-1^. Hydrogen uptake during the reduction was continuously recorded using a thermal conductivity detector (TCD).

The oxygen vacancies formation energy at nearly active sites was calculated using DFT. The model was constructed based on the rock-salt structure of NaCl. A NaCl (100) surface was first cleaved, with all sodium atoms replaced by metal elements and all chloride atoms replaced by oxygen. Random structures were then generated using a Python script, and DFT calculations were performed to obtain the energies of all configurations. For this study, the most stable surface structures of both Li-HEO and HEO were selected. Oxygen vacancies were subsequently introduced on the surface, and the corresponding formation energies were calculated. The reported values represent the averages of vacancy formation energies at the nearby Cu, Co and Ni metal sites.

Density functional theory calculations were performed using the Vienna ab initio simulation package (VASP) with periodic boundary conditions. The projector augmented wave (PAW) method and the Perdew-Burke-Ernzerhof (PBE) functional under the generalized gradient approximation (GGA) were applied. The kinetic energy cut-off was set to 450 eV, and the force threshold in structure optimization was 0.05 eV/Å, the convergence criteria for energy is set as 10^-5^ eV. A 3 x 3 x 1 k-point mesh was used in calculation. The climbing image nudged elastic band (CI-NEB) method was employed to determine reaction transition states. The onsite Coulomb correction (U-J = 6.2 eV) is incorporated to depict the localized electronic states of Ni and Co 3d in mixed valence metals. Dispersion forces were included with DFT-D3 method. Spin polarization was enabled for all calculations.

The catalyst model consisted of a 12-atom Cu pyramidal nanocluster supported on a LiHEO (LiNiMgCuZnCoO_x_) high-entropy oxide slab, as well as a corresponding Li-free (NiMgCuZnCoO_x_) slab. The slabs were constructed using a 4 × 4 supercell with six atomic layers and were oriented along the (001) direction, with a vacuum layer exceeding 15 Å to avoid spurious interactions between periodic images. Periodic boundary conditions were applied in all three dimensions.

During structural optimization, the bottom four layers were fixed to emulate a semi-infinite bulk, while the Cu cluster, the top two surface layers, and all adsorbed species were fully relaxed.

Adsorption energies were calculated relative to the bare slab and the gas-phase reference according to

E_ads = E(slab + X*) − E(slab) − E(X_gas).

Reaction energies for the elementary hydrogenation step

C_2_H_4_* + H_2_* → C_2_H_5_* + H*

were computed as ΔErxn = E(FS) − E(IS), based on the optimized co-adsorbed initial and final states.

*The Inelastic neutron scattering (INS)* spectra was obtained by placing the catalysts in 6 mm V cans and collecting data for 1 h at 10 K. These measurements were carried out at ORNL’s Spallation Neutron Source under the Scientific User Facilities Division. Beamtime was granted on VISION (BL-16), with the backscattering detector employed for data collection.

*H_2_-D_2_ exchange* measurements were conducted on an AMI-300 instrument equipped with a mass spectrometer (MS, Pfeiffer Vacuum). About 10 mg sample was loaded and pretreated H_2_ flow (30 mL·min^-1^) at 300 °C for 1 h, followed by cooling to 50 °C under He flow. The gas feed was then switched to a mixed flow of H_2_ (20 mL·min^-1^) and D_2_ (20 mL·min^-1^), and heating was performed from 50 to 600 °C at 10 °C·min^-1^. The HD signal (m/z=3) was continuously monitored by MS. The HD signal intensity was normalized to the signal value at 600 °C, and the onset and T_50_ temperatures for HD formation were compared to evaluate hydrogen activation performance.

*Ethylene pulse hydrogenation experiments* were performed in a fixed-bed quartz reactor using 10 mg of catalyst. The catalyst was pretreated under H_2_ at 300 °C for 1 h and then cooled to the 100 °C under a He atmosphere. Ethylene pulses were introduced into a 4% H_2_/Ar carrier gas stream at atmospheric pressure. The effluent gases were analyzed online by a quadrupole mass spectrometer, with m/z = 26 and m/z = 30 assigned to ethylene and ethane, respectively. The MS signals were integrated to obtain peak areas, and the relative hydrogenation behavior was evaluated using the R value defined as R = A(C_2_H_4_)/A(C_2_H_6_). A higher R value indicates suppressed over-hydrogenation of ethylene.

*In situ diffuse reflectance infrared Fourier transform spectroscopy (DRIFTS)* measurements were carried out on Nicolet iS50 Spectrometer (Thermo Fisher Scientific) to investigate acetylene adsorption and surface intermediates under hydrogenation conditions. Prior to measurements, the catalyst sample was pretreated in flowing 5%H_2_/He at 300 °C for 1 h and subsequently cooled to the 100 °C under inert gas. A gas mixture containing 1%C_2_H_2_ and 5%H_2_ (balanced with He) was introduced into the DRIFTS cell at a total flow rate of 30 mL·min^-1^. Spectra were collected after 30 min under reaction conditions. He purging experiments were conducted by switching the feed to pure He while maintaining the same temperature.

## **Acetylene semi-hydrogenation performance evolution**

Evaluation of the acetylene semi-hydrogenation catalytic performance was conducted in a fixed-bed reactor under ambient pressure. For the performance tests at different temperatures, 200 mg of catalyst was placed in a quartz reactor and mixed with about 200 mg of inert quartz. Prior to reaction, the catalysts were pretreated under a flow of 5% H_2_ in Ar at the designated reduction temperature for 1 h and then cooled to the reaction temperature. Li-doped HEO catalysts reduced at different temperatures, denoted as LiHEO-200R, LiHEO-300R, and LiHEO-400R, were tested at 100 °C to examine the effect of reduction temperature. The gas feed was switched to the reaction mixture composed of 1%C_2_H_2_/Ar and 5%H_2_/Ar with a volume ratio of 1:1 (total flow 30 mL·min^-1^). In addition, LiHEO-300R and the undoped HEO-300R were tested over a reaction temperature range from 60 to 100 °C. Stability test was performed at 100 °C with a constant feed gas composition and a fixed total flow rate corresponding to a WHSV of 15000 mL·g_cat_^-1^·h^-1^.

The effluent gas was analyzed online using SRI gas chromatography equipped with a TCD detector to monitor C_2_H_2_ conversion and C_2_H_4_ and C_2_H_6_ selectivity. The C_2_H_2_ conversion and C_2_H_4_ and C_2_H_6_ selectivity were calculated as:

| $\begin{aligned} X_{{CO}_{2}}=\frac{M_{C_{2}H_{2}, in}-M_{C_{2}H_{2}, out}}{M_{C_{2}H_{2}, in}}\times100\% \end{aligned}$ | (1) |
| --- | --- |
| $S_{C_{2}H_{4}}=\frac{M_{C_{2}H_{4},out}}{M_{C_{2}H_{2}, in}-M_{C_{2}H_{2}, out}}\times100\%$ | (2) |
| $S_{C_{2}H_{6}}=\frac{M_{C_{2}H_{6},out}}{M_{C_{2}H_{2}, in}-M_{C_{2}H_{2}, out}}\times100\%$ | (3) |

Wherein $M_{C_{2}H_{2}, in}$and $M_{C_{2}H_{2}, out}$ are the molar fraction of C_2_H_2_ in the feed and effluent gas, $M_{C_{2}H_{4},out}$ and $M_{C_{2}H_{6},out}$ present the molar concentration of C_2_H_4_ and C_2_H_6_ in the product gas, respectively.

Supplementary Figures

**
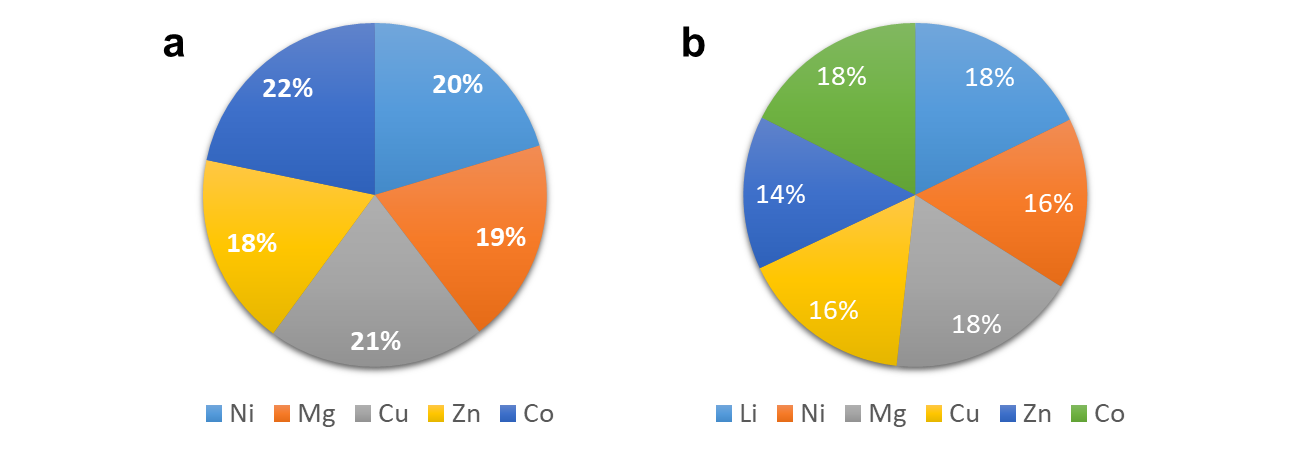
**

Figure S1. Molar fractions of individual metals in HEO (a) and LiHEO (b) obtained from ICP measurements.

**
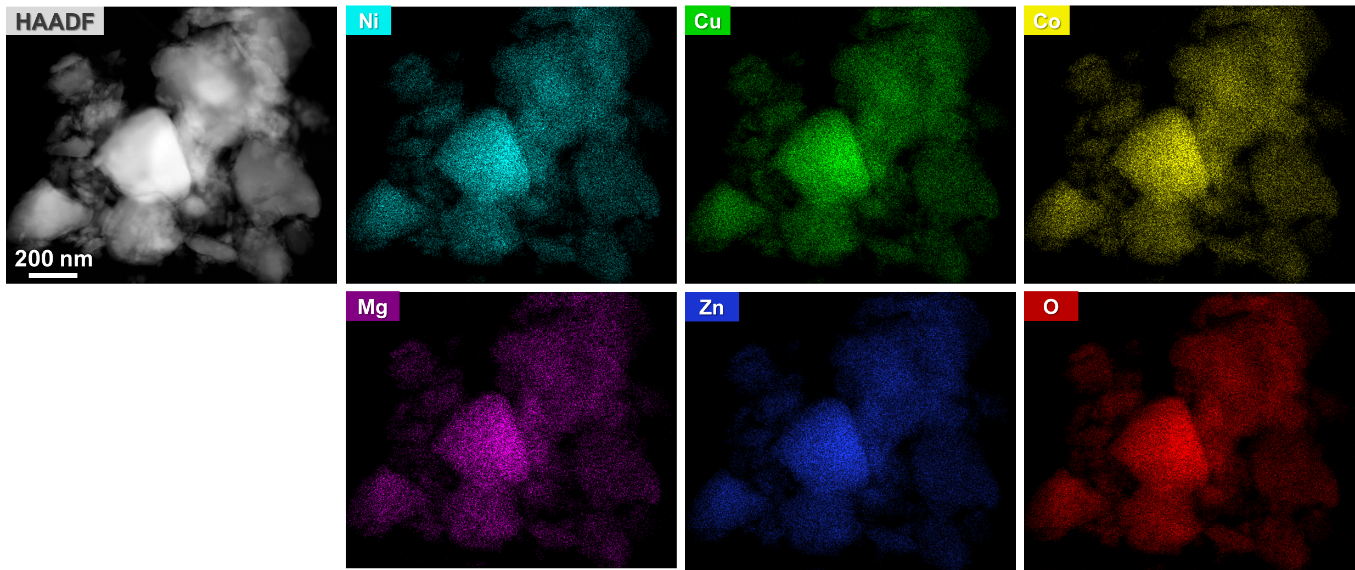
**

Figure S2. HAADF-STEM-EDS mapping results of fresh LiHEO.

**
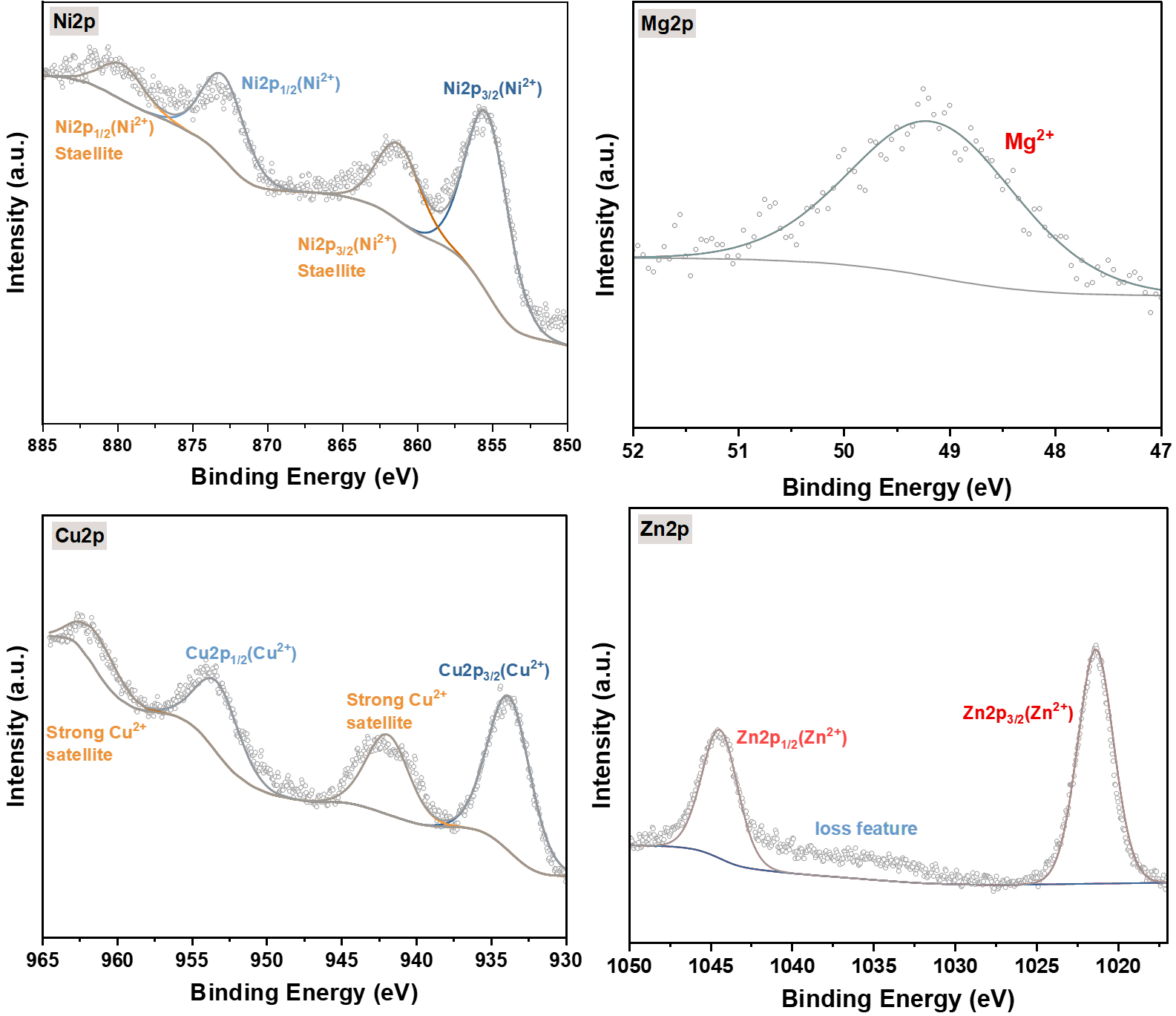
**

Figure S3. Ni 2p, Mg 2p, Cu 2p, and Zn 2p XPS spectra of fresh LiHEO.

**
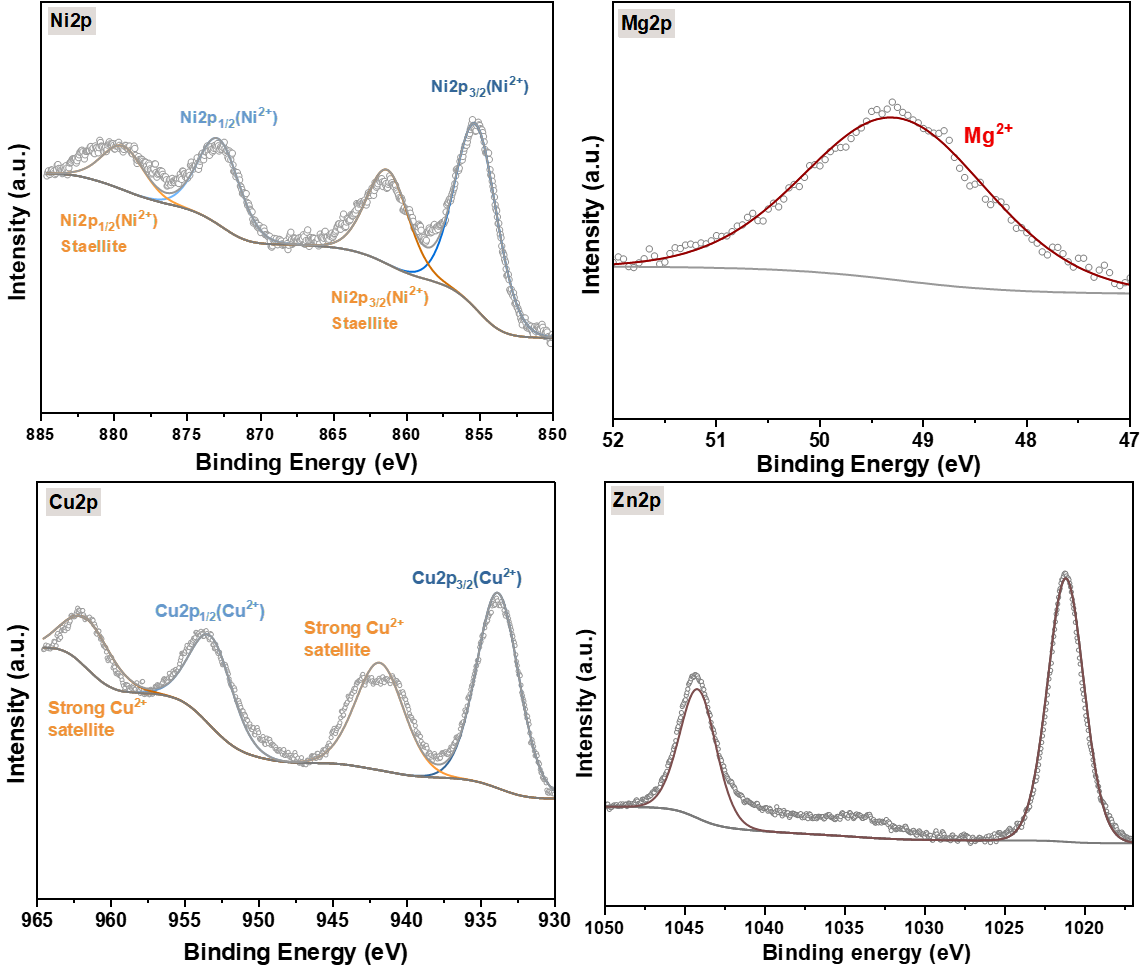
**

Figure S4. Ni 2p, Mg 2p, Cu 2p, and Zn 2p XPS spectra of fresh HEO.


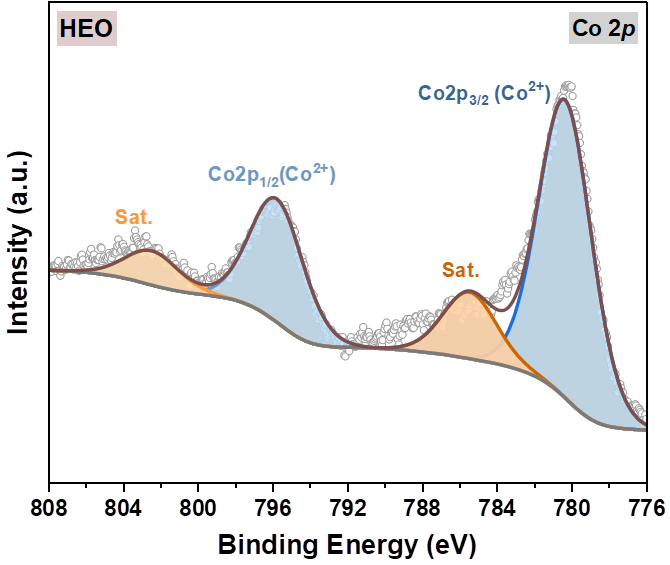


Figure S5. Co 2p XPS spectra of fresh HEO.

**
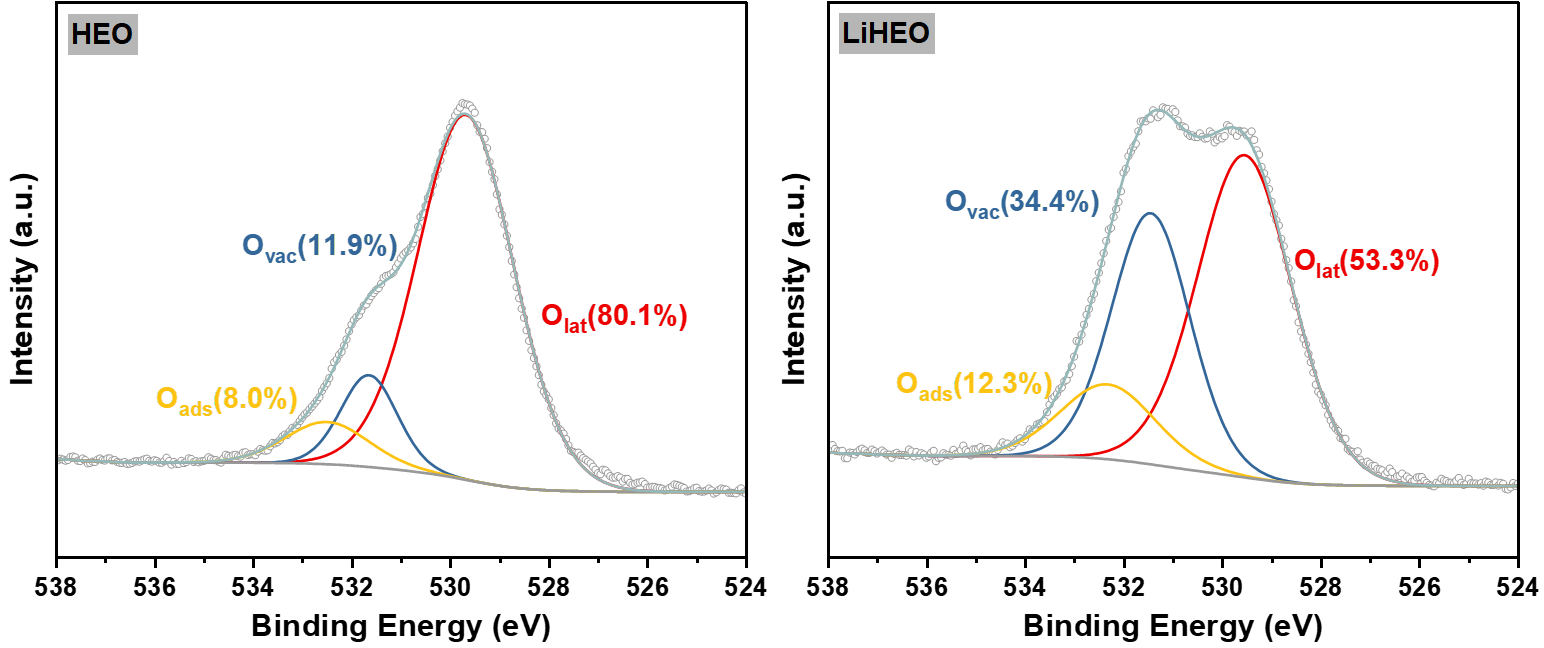
**

Figure S6. O 1s XPS spectra of fresh HEO and LiHEO samples.

Figure S7. Co K-edge XANES spectra of the fresh LiHEO sample.

**
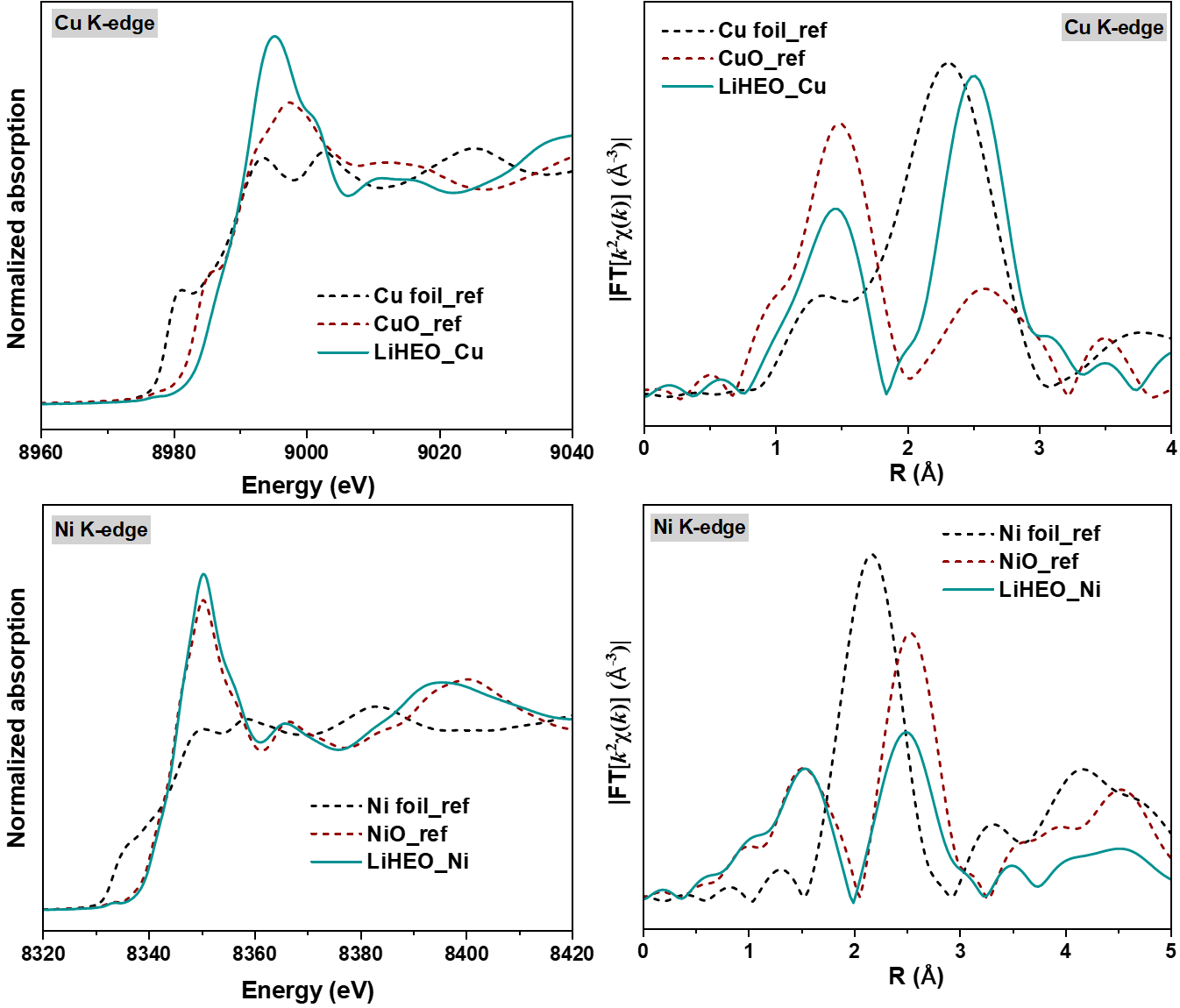
**

Figure S8. Cu and Ni K-edge XANES and EXAFS spectra of the fresh LiHEO sample.

Figure S9. H_2_-TPR profiles of HEO and LiHEO samples.

Figure S10. In situ HTXRD patterns of HEO (a) and LiHEO (b) collected from 25 to 500 °C under a 2%H_2_/N_2_ atmosphere.

Figure S11. NAP-XPS spectra of HEO and LiHEO for the Cu LMM regions collected from 25 to 500°C.

**
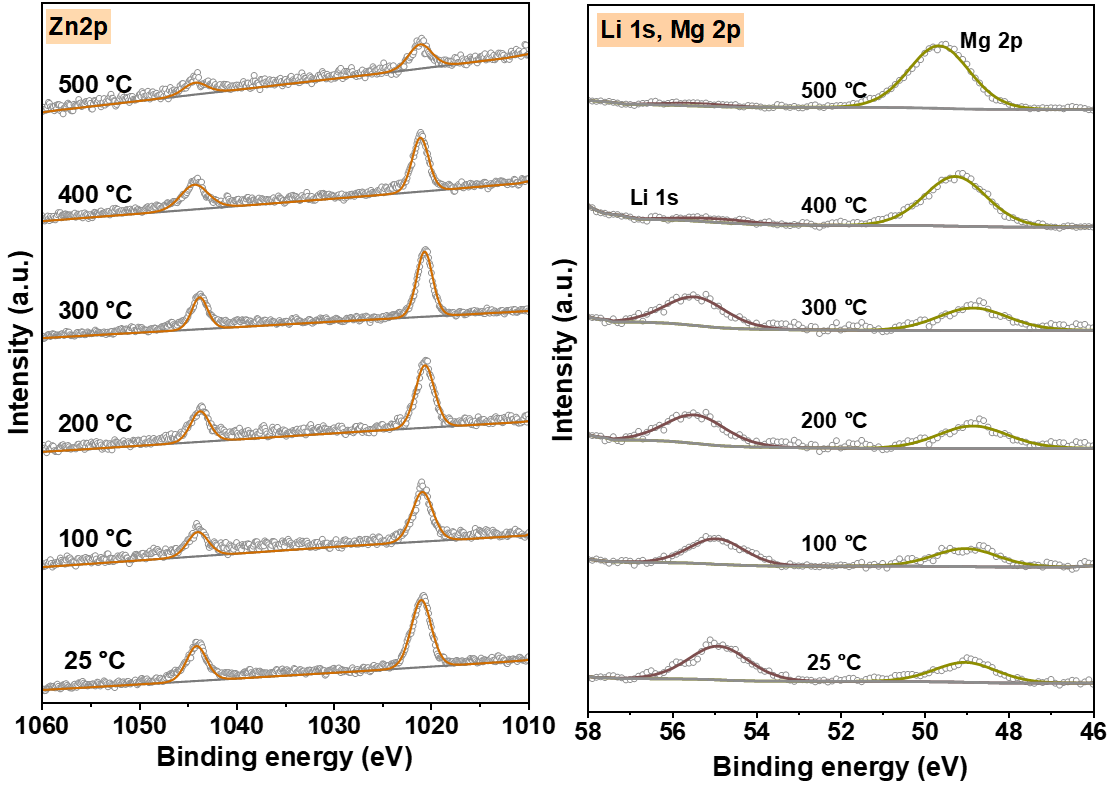
**

Figure S12. NAP-XPS spectra of LiHEO for the Zn 2p, Li 1s, and Mg 2p regions collected from 25 to 500°C.

Figure S13. The DFT model structures of (a) HEO and (b) LiHEO. (c) The average oxygen vacancy formation energy of Cu, Ni, and Co sites.

Figure S14. (a) XRD patterns of the Cu/MgO–ZnO reference catalyst before and after reduction in 5% H_2_/Ar at 300 °C, showing the transformation of CuO to metallic Cu.; (b) comparison of acetylene conversion over LiHEO-300R and Cu/MgO–ZnO-300R under identical conditions, highlighting the negligible activity of the reference catalyst.(1 atm, 2.5%H_2_/0.5%C_2_H_2_/Ar, 15000 mL·g^-1^·h^-1^).

Figure S15. Elements composition analysis results of spent HEO-300R (a) and spent LiHEO-300R (b) samples.

Figure S16. HAADF-STEM-EDS mapping images of LiHEO-300R sample.

**
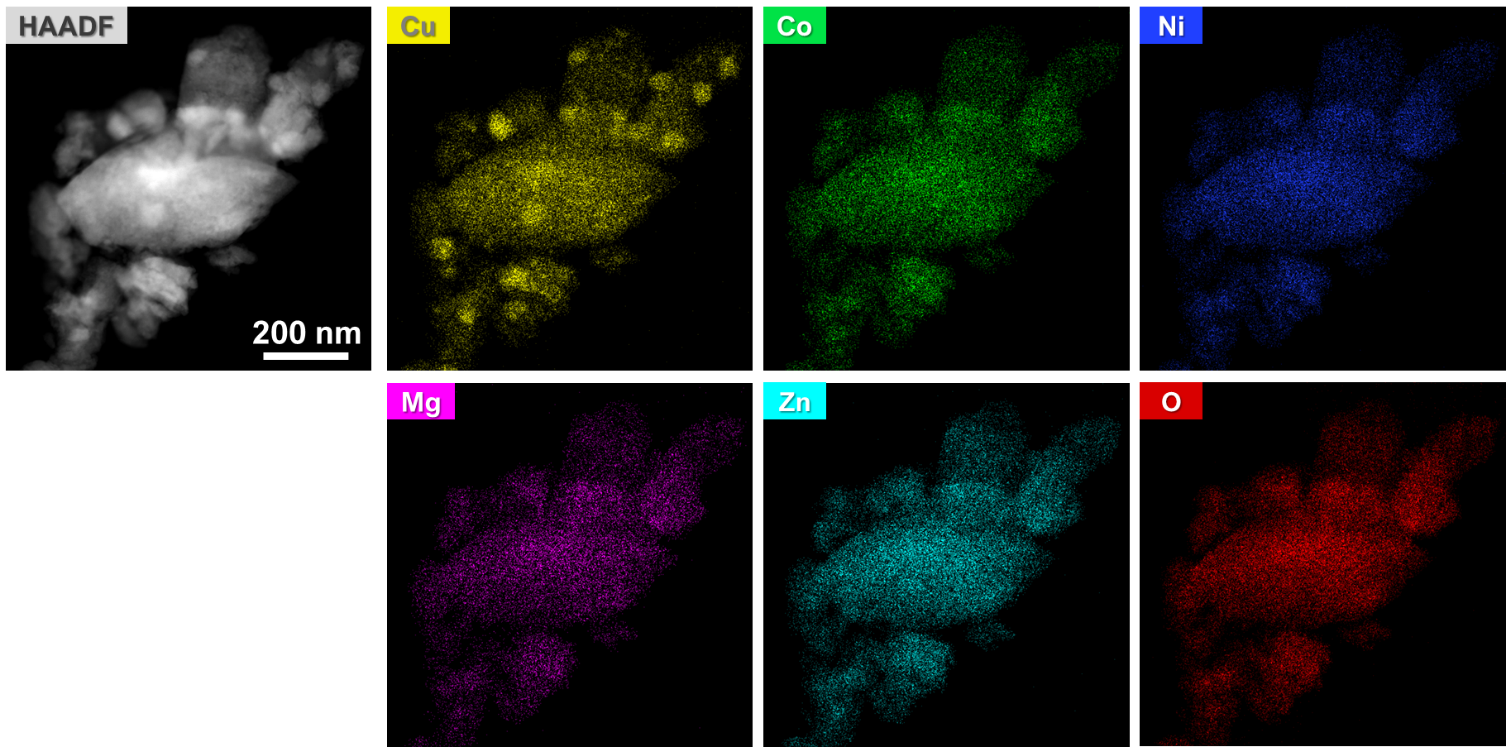
**

Figure S17. HAADF-STEM-EDS mapping images of HEO-300R sample.

**
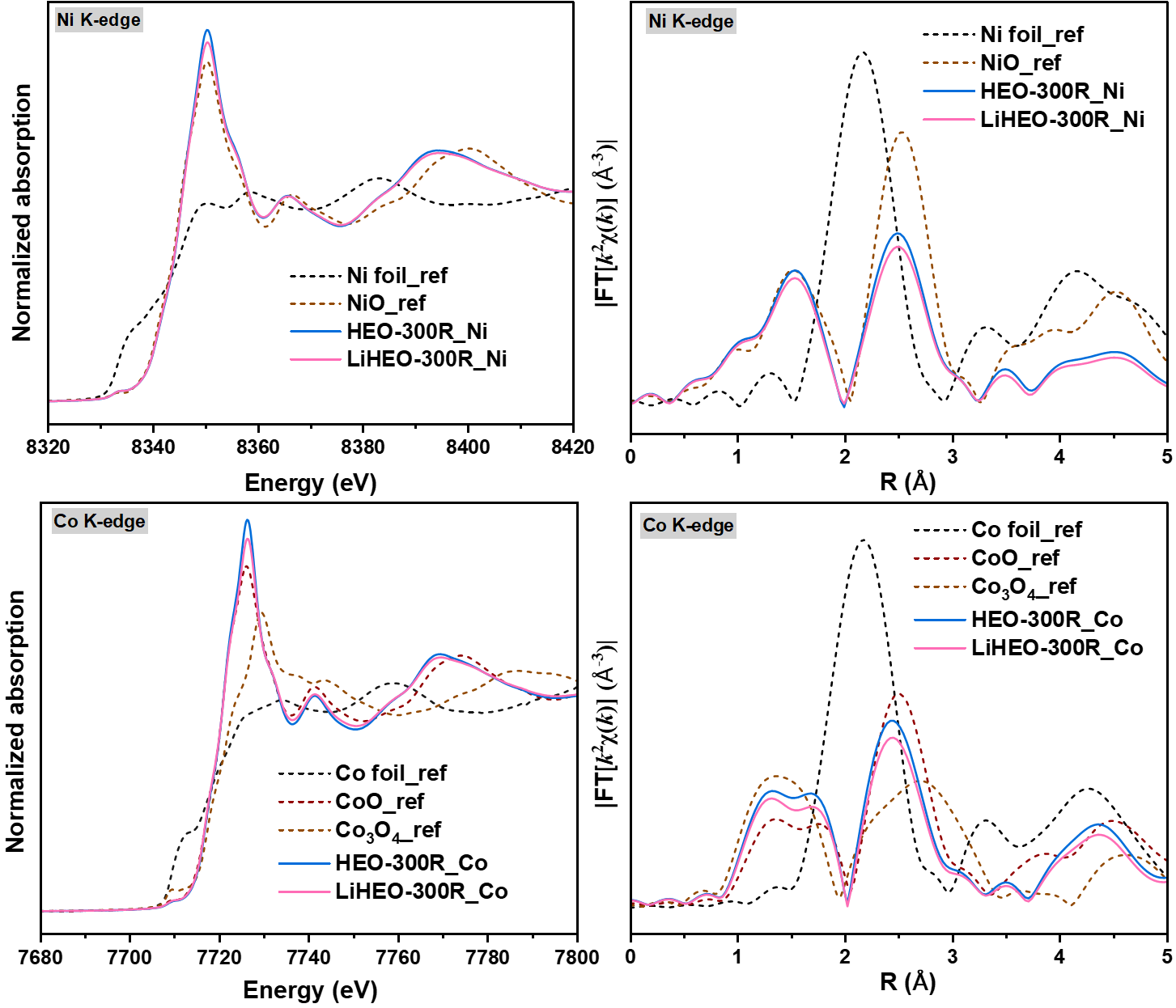
**

Figure S18. Ni and Co K-edge XANES and EXAFS spectra of HEO-300R and LiHEO-300R samples.

**
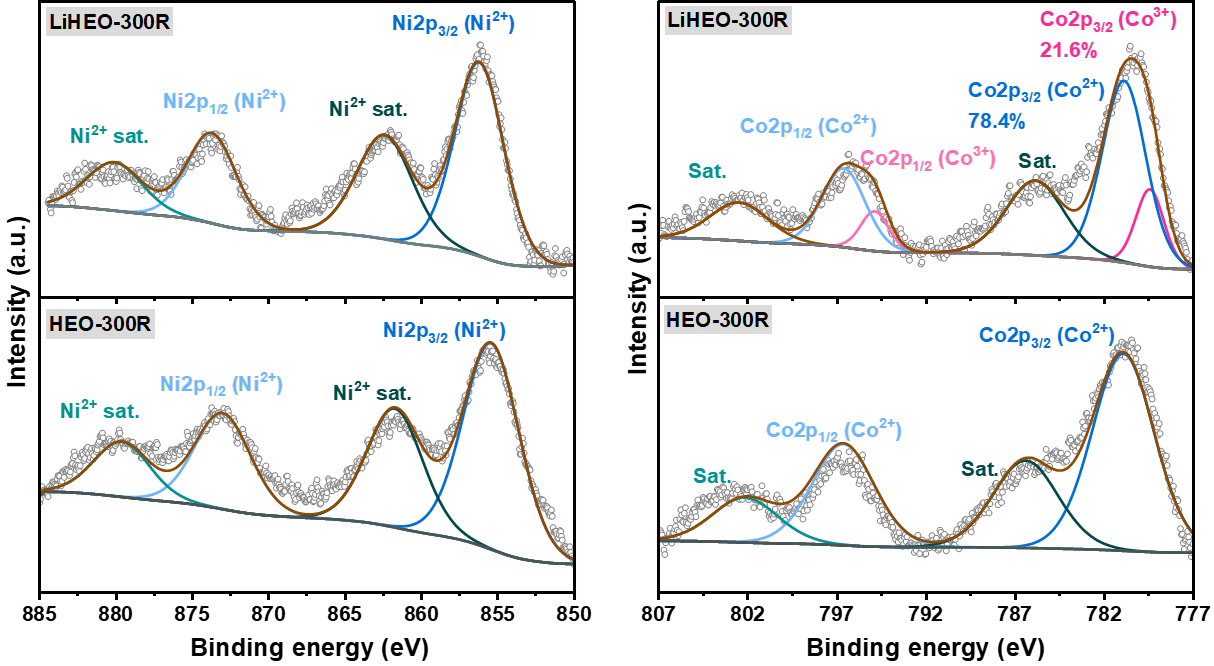
**

Figure S19. XPS spectra of the Ni 2p and Co 2p regions for HEO-300R and LiHEO-300R samples.

Figure S20. XPS spectra of the Zn 2p, Mg 1s for HEO-300R (a, b) and Zn 2p and Mg 2p regions for LiHEO-300R (c, d).

Figure S21. O 1s XPS spectra of HEO-300R (a) and LiHEO-300R (b) samples.

**
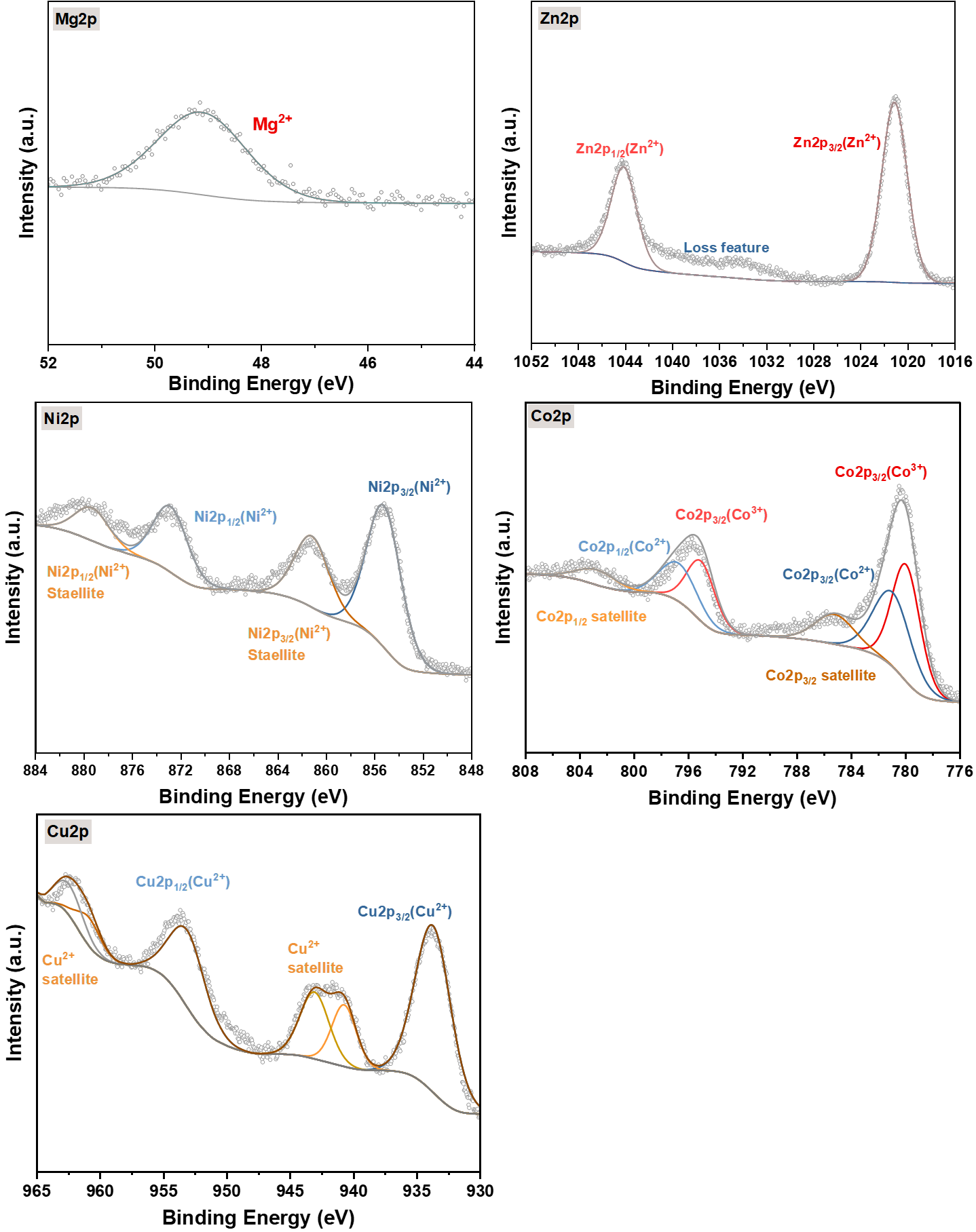
**

Figure S22. XPS spectra of LiHEO-200R sample.

**
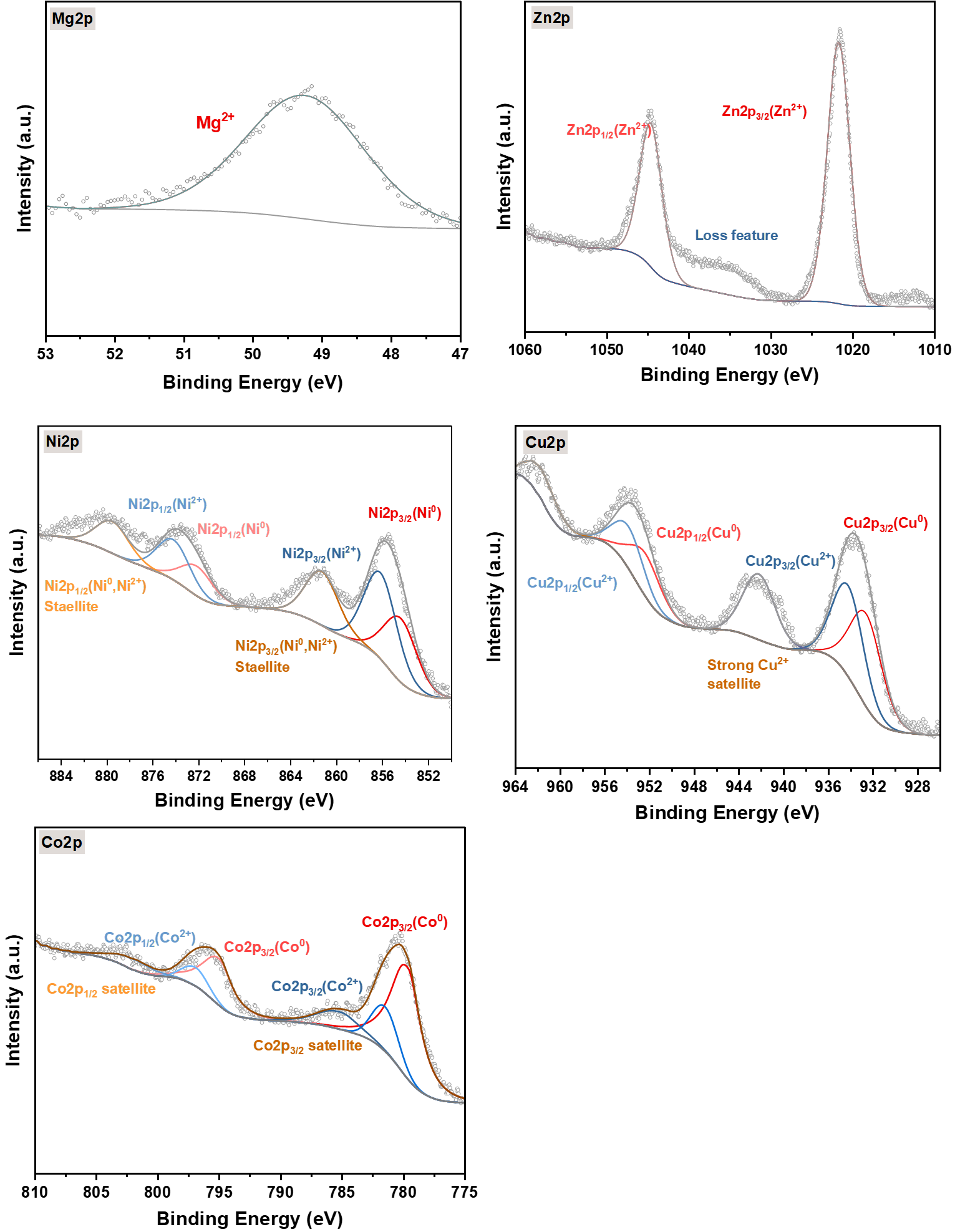
**

Figure S23. XPS spectra of LiHEO-400R sample.

**
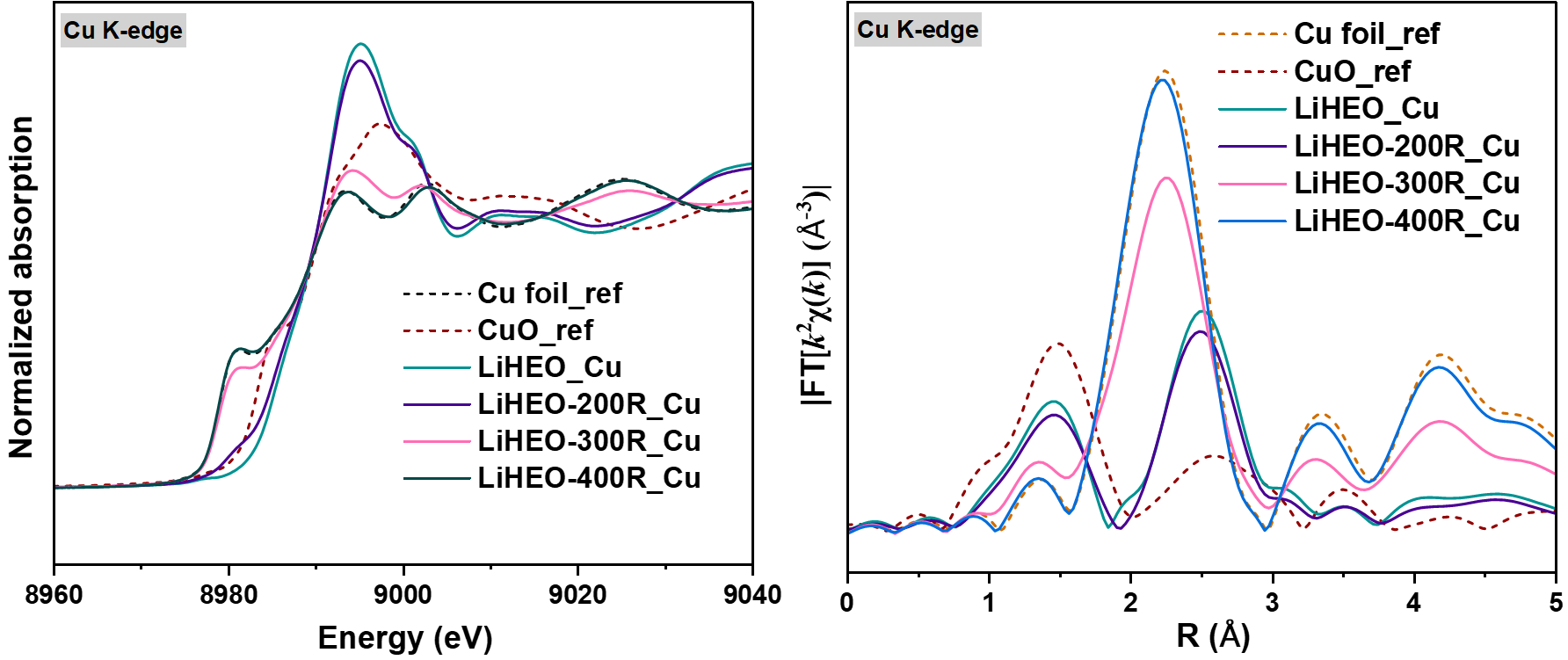
**

Figure S24. Cu K-edge XANES and EXAFS spectra of LiHEO, LiHEO-200R, LiHEO-300R, and LiHEO-400R samples.

**
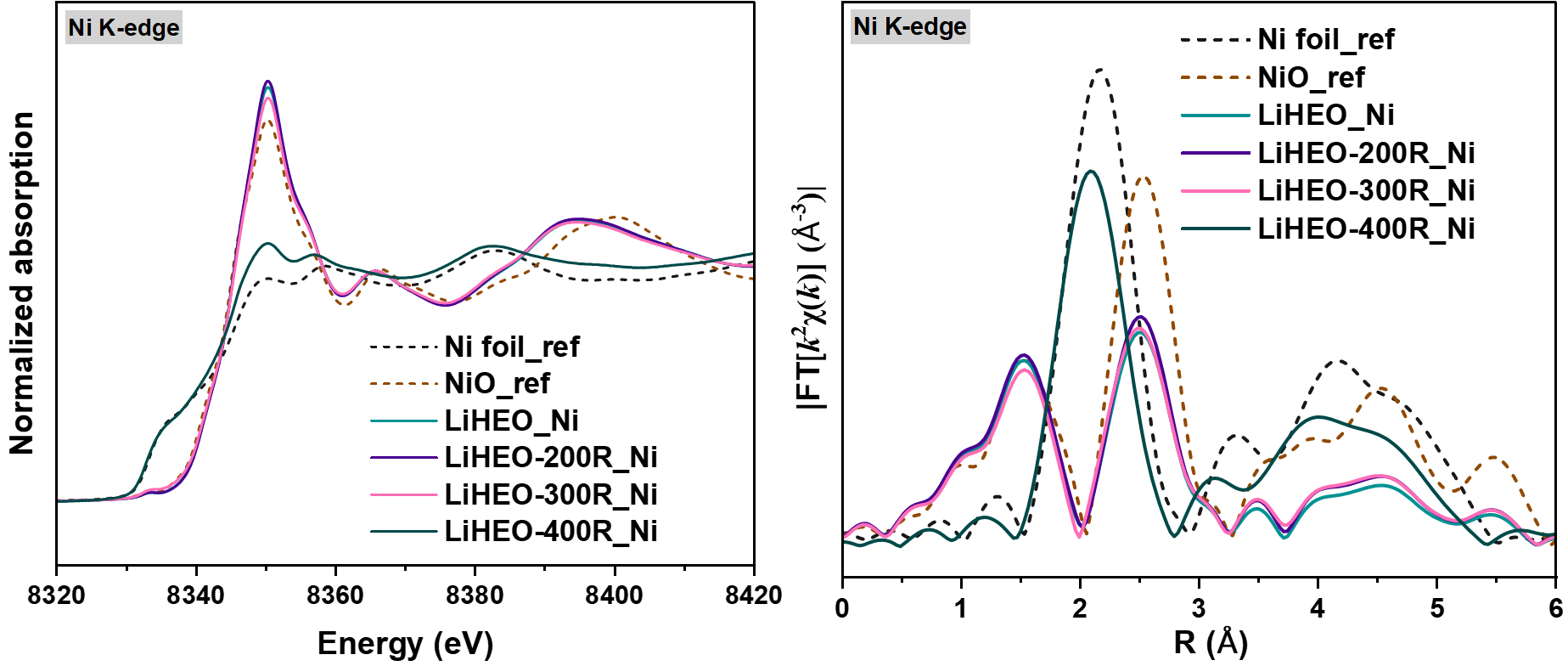
**

Figure S25. Ni K-edge XANES and EXAFS spectra of LiHEO, LiHEO-200R, LiHEO-300R, and LiHEO-400R samples.

**
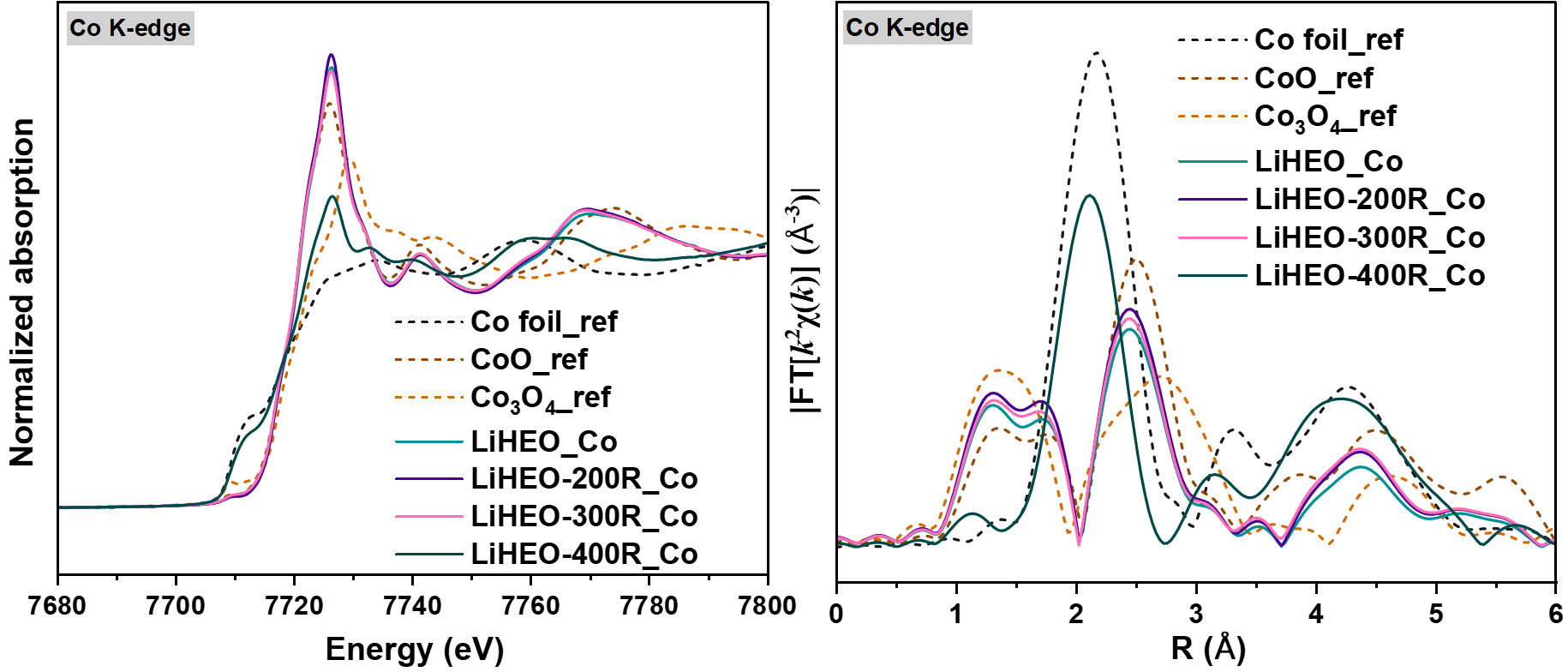
**

Figure S26. Co K-edge XANES and EXAFS spectra of LiHEO, LiHEO-200R, LiHEO-300R, and LiHEO-400R samples.

Figure S27. XRD patterns of spent HEO and LiHEO samples, showing that the main crystalline features are largely preserved, with a weak shoulder at ~44° attributed to metallic species formed during reduction.

**
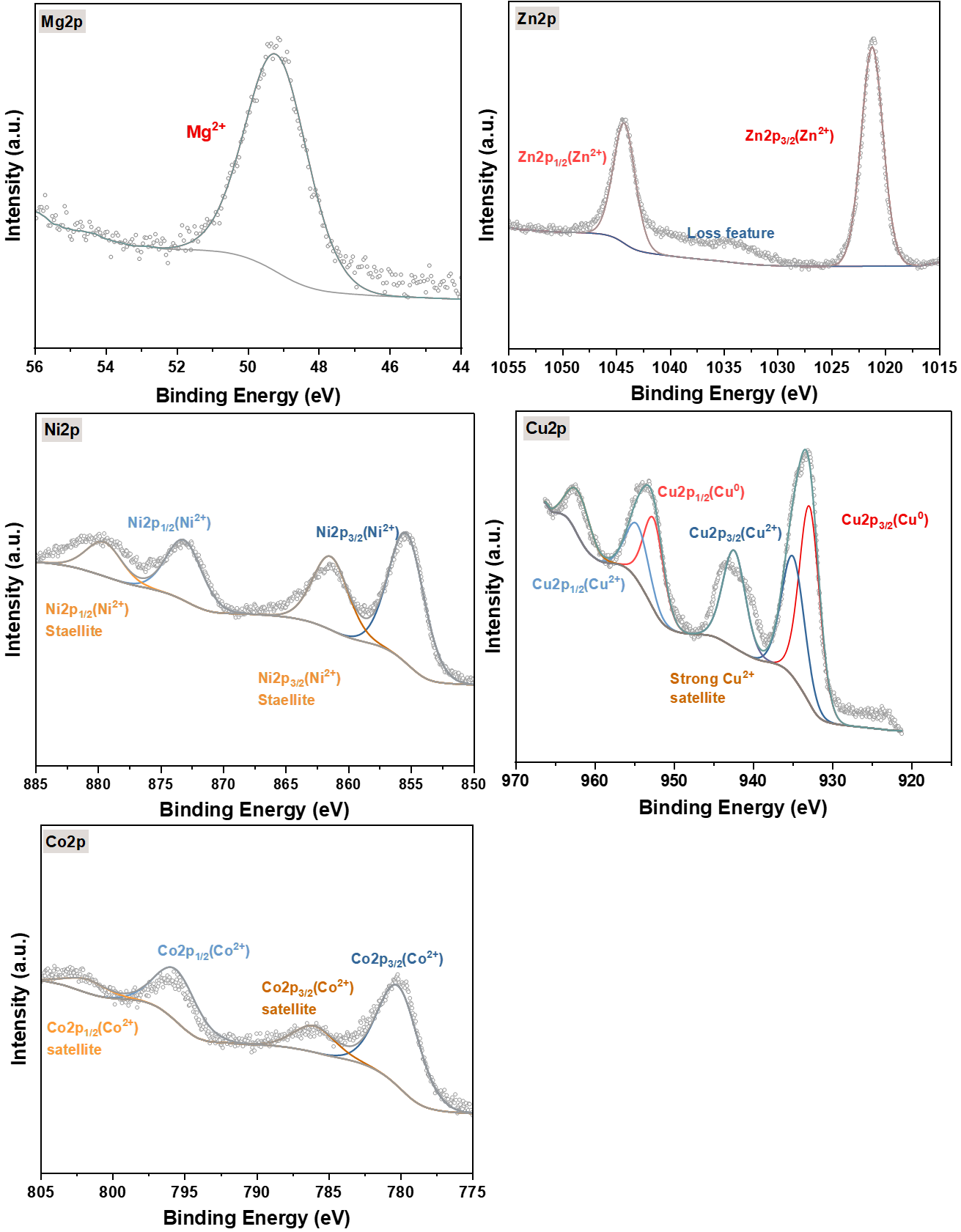
**

Figure S28. XPS spectra of spent HEO sample, indicating that Cu remains predominantly in the metallic state, while Mg, Zn, Ni, and Co are largely retained in their oxidized states.


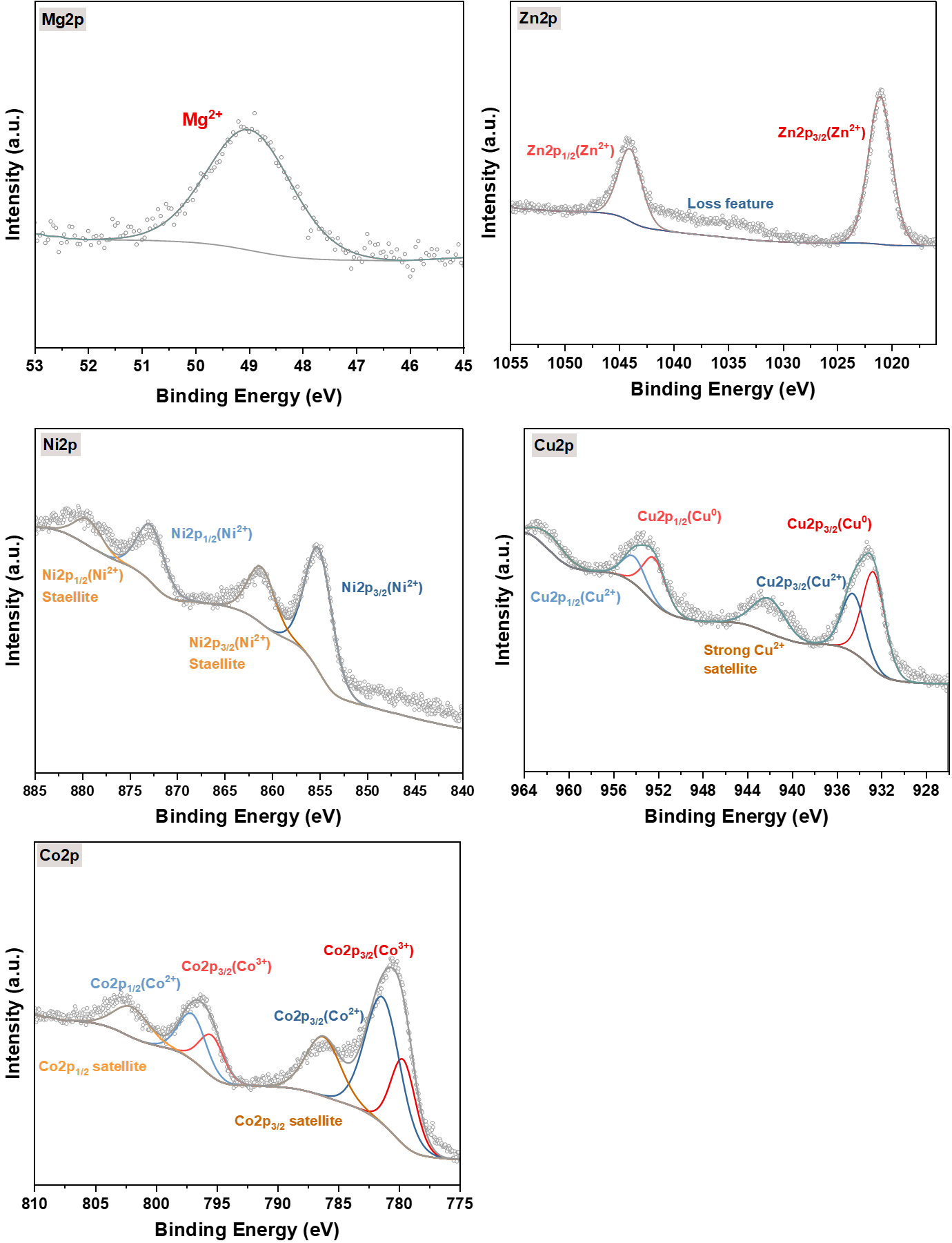


Figure S29. XPS spectra of spent LiHEO sample, indicating that Cu remains predominantly in the metallic state, while Mg, Zn, Ni, and Co are largely retained in their oxidized states.

Figure S30. In situ DRIFTS spectra of HEO and LiHEO collected under C_2_H_2_ hydrogenation conditions and after He purging.

Figure S31. INS spectra of fresh and reduced HEO and LiAl-HEO compared to reference samples.

Figure S32. (a) DFT models of exsolved Cu clusters on LiHEO-300R (left) and HEO-300R (right) surfaces, and (b) the corresponding reaction energy profiles for C_2_H_2_ hydrogenation.

Table S1 Standard reduction potential of different metal cations.

| **Metal cations** | **Standard reduction potential (E^0^)** |
| --- | --- |
| Cu^2+^ | +0.34 V |
| Co^3+^ | +1.82V |
| Co^2+^ | -0.28 V |
| Ni^2+^ | -0.25 V |
| Zn^2+^ | -0.76 V |
| Mg^2+^ | -2.37 V |

Table S2. Relative contents of fitted NAP-XPS spectra for Cu 2p, Ni 2p, and Co 2p for LiHEO sample. Numbers indicate peak positions (in eV), and values in parentheses represent the corresponding fractions.

| Temperature  (°C) | Cu2p | | Ni2p | | Co2p | | |
| --- | --- | --- | --- | --- | --- | --- | --- |
|  | Cu^2+^ | Cu^0^ | Ni^2+^ | Ni^0^ | Co^3+^ | Co^2+^ | Co^0^ |
| 25 | 933.44 (100%) | / | 854.83  (100%) | / | 779.60  (50.5%) | 780.96  (49.5%) | / |
| 100 | 933.44 (100%) | / | 854.83  (100%) | / | 779.60  (50.5%) | 780.96  (49.5%) | / |
| 200 | 933.44 (50.8%) | 931.97  (49.2%) | 854.83  (100%) | / | 779.40  (45.3%) | 780.96  (54.7%) | / |
| 300 | 933.44  (22.6%) | 931.97  (77.4%) | 854.83  (80.2%) | 852.53  (19.8%) | 779.60  (15.1%) | 780.96  (52.7%) | 778.4  (32.2%) |
| 400 | 933.44  (16.9%) | 931.97  (83.1%) | 854.83  (59.0%) | 852.56  (41.0%) | / | 780.96  (49.1%) | 778.4  (50.9%) |
| 500 | 933.44  (15.6%) | 931.97  (84.4%) | 854.83  (34.3%) | 852.56  (65.7%) | / | 780.96  (35.8%) | 778.4  (64.2%) |

Table S3. Relative contents of fitted NAP-XPS spectra for Cu 2p, Ni 2p, and Co 2p for HEO sample. Numbers indicate peak positions (in eV), and values in parentheses represent the corresponding fractions.

| Temperature  (°C) | Cu2p | | Ni2p | | Co2p | |
| --- | --- | --- | --- | --- | --- | --- |
|  | Cu^2+^ | Cu^0^ | Ni^2+^ | Ni^0^ | Co^2+^ | Co^0^ |
| 25 | 933.79 (100%) | / | 855.40  (100%) | / | 780.88  (100%) | / |
| 100 | 933.79 (100%) | / | 855.4  (100%) | / | 780.88  (100%) | / |
| 200 | 933.79 (68.2%) | 932.59  (31.8%) | 855.4  (100%) | / | 780.88  (100%) | / |
| 300 | 933.79  (22.0%) | 932.50  ( 78.0%) | 855.4  (69.9%) | 852.47  (30.1%) | 780.88  (86.6%) | 778.4  (13.4%) |
| 400 | 933.79  (24.3%) | 932.59  (75.7%) | 855.4  (53.5%) | 852.47  (46.5%) | 780.88  (76.3%) | 778.4  (23.7%) |
| 500 | 933.79  (22.7%) | 932.59  (77.3%) | 855.4  (44.7%) | 852.72  (55.3%) | 780.88  (65.8%) | 778.4  (34.2%) |

Table S4. Comparison of catalytic performance (e.g., conversion, selectivity) of the present catalysts with previously reported transition metal-based and noble metal-based catalysts.

| No. | Catalyst | Catalyst amount (mg) | | Feed (vol.%) | | WHSV  (mL·g^-1^·h^-1^) | | T | | Conv. | | Sele. | | Reaction rate  (mmol_C2H2_·g^-1^·h^-1^) | | Ref. |  |
| --- | --- | --- | --- | --- | --- | --- | --- | --- | --- | --- | --- | --- | --- | --- | --- | --- | --- |
|  |  |  |  | (C_2_H_2_:H_2_:C_2_C_4_) | |  |  | (ºC) | | (%) | | (%) | |  |  |  |  |
| / | LiHEO-300R | 200 | | 0.5:2.5:0, in Ar | | 15000 | | 100 | | 100 | | 100 | | 3.35 | | This work |  |
| 1 | Cu1/ND@G | 200 | | 1:10:20, in He | | 3000 | | 200 | | ＞95 | | ＞98 | | 1.27 | | ^[2]^ |  |
| 2 | Cu_1_/Al_2_O_3_ | 150 | | 1:10:50, in Ar | | 8000 | | 188 | | 100 | | 91 | | 3.57 | | ^[3]^ |  |
| 3 | Cu/Fe_0.16_MgO_x_ | 200 | | 0.33:1.02:32.86, in N_2_ | | 10032 | | 215 | | 100 | | 95 | | 1.48 | | ^[4]^ |  |
| 4 | Cu_1_/g-C_3_N_4_ | 150 | | 0.5:5:25, in Ar | | 12000 | | 260 | | 10 | | 60 | | 0.27 | | ^[5]^ |  |
| 5 | Ni_1_Cu_2_/g-C_3_N_4_ | 150 | | 0.5:5:25, in Ar | | 12000 | | 170 | | 100 | | 90 | | 2.68 | | ^[5]^ |  |
| 6 | Cu1/ND@NG | 100 | | 1:10:20, in He | | 6000 | | 200 | | ＜20 | | ＞96 | | 0.54 | | ^[6]^ |  |
| 7 | Cu/γ-Al_2_O_3_ | 300 | | 0.31% C_2_H_2_/30.4% C_2_H_4_/1.0 % C_3_H_8_/ 3.1% H_2_, in N_2_ | | 9780 | | 200 | | 100 | | 80 | | 1.35 | | ^[7]^ |  |
| 8 | CuZn/NC2 | 350 | | 0.33:0.66:33, in N_2_ | | 6000 | | 180 | | 97 | | 97.5 | | 0.86 | | ^[8]^ |  |
| 9 | CuZn-Co/HEOs | 200 | | 0.71% C_2_H_2_/70.72% C_2_H_4_/ 2.86% H_2_, in N_2_ | | 3800 | | 200 | | ~100 | | 95.7 | | 1.20 | | ^[9]^ |  |
| 10 | [Na-Ni@CHA](mailto:Na-Ni@CHA) | 200 | | 1:16:0, in He | | 15000 | | 180 | | 100 | | 97 | | 6.70 | | ^[10]^ |  |
| 11 | NiCuFeGaGe/ SiO_2_ | 100 | | 1:10:10, in He | | 30000 | | 220 | | 100 | | 93 | | 13.39 | | ^[11]^ |  |
| 12 | (Ni_0.8_Cu_0.2_)_3_Ga/TiO_2_ | 80 | | 1:10:0, in He | | 37500 | | 150 | | 100 | | 96 | | 16.74 | | ^[12]^ |  |
| 13 | NiIn/MgAl | 200 | | 0.5:2.5:30, in N_2_ | | 9000 | | 110 | | 100 | | 96.3 | | 2.01 | | ^[13]^ |  |
| 14 | AgPd_0.01_/SiO_2_ | 30 | | 1:20:20, in He | | 60000 | | 160 | | ＞90 | | ＞80 | | 24.11 | | ^[14]^ |  |
| 15 | Pd-In/Al_2_O_3_ | 50 | | 0.87:3.1:73, in N_2_ | | 144022 | | 120 | | 99 | | 77 | | 55.38 | | ^[15]^ |  |
| 16 | Pd_1_/C_3_N_4_ | 50 | | 0.5:1:25, in Ar | | 60000 | | 115 | | 99 | | 83 | | 13.26 | | ^[16]^ |  |
| 17 | CuPd_0.006_/SiO_2_ | 30 | | 1:20:20, in He | | 60000 | | 160 | | 100 | | 85 | | 26.79 | | ^[17]^ |  |
| 18 | Pd_1_/ND@G | 30 | | 1:10:20, in He | | 60000 | | 180 | | 100 | | 90 | | 26.79 | | ^[18]^ |  |
| 19 | Pd-SAs-900 | 1000 | | 0.5:5:50, in He | | 1200 | | 120 | | 96 | | 93 | | 0.26 | | ^[19]^ |  |
| 20 | ISA-Pd/MPNC | 200 | | 0.5:5:50, in He | | 10500 | | 110 | | 83 | | 82 | | 1.95 | | ^[20]^ |  |
| 21 | Pd_1_@Cu-SiW | 300 | | 0.5:5:50, in He | | 4000 | | 120 | | 98 | | 92 | | 0.88 | | ^[21]^ |  |
| 22 | Pd_1_Cu_1_/ND@G | 30 | | 1:10:20, in He | | 60000 | | 110 | | 100 | | 92 | | 26.79 | | ^[22]^ |  |
| 23 | Cu/B2 CuPd | 20 | | 0.5:3:0, in Ar | | 150000 | | 90 | | 100 | | 95.2 | | 33.48 | | ^[23]^ |  |
| 24 | Pd-STO | 20 | | 1:10:20, in Ar | | 120000 | | 100 | | 98 | | 92 | | 52.50 | | ^[24]^ |  |
| 25 | Ru_1_Cu_n_/SiO_2_ | 100 | | 1:10:20, in He | | 6000 | | 170 | | 100 | | 97.6 | | 2.68 | | ^[25]^ |  |
| 26 | Pd_1_/SiO_2_-NH_2_ | | 5 | | 1:10:20, in He | | 240000 | | 190 | | 100 | | 92 | | 107.14 | ^[26]^ | |

References

[1] B. Ravel, M. Newville, *J. Synchrotron Radiat.* **2005**, *12*, 537-541.

[2] F. Huang, Y. Deng, Y. Chen, X. Cai, M. Peng, Z. Jia, J. Xie, D. Xiao, X. Wen, N. Wang, *Nat. Commun.* **2019**, *10*, 4431.

[3] X. Shi, Y. Lin, L. Huang, Z. Sun, Y. Yang, X. Zhou, E. Vovk, X. Liu, X. Huang, M. Sun, *ACS Catal.* **2020**, *10*, 3495-3504.

[4] F. Fu, Y. Liu, Y. Li, B. Fu, L. Zheng, J. Feng, D. Li, *ACS Catal.* **2021**, *11*, 11117-11128.

[5] J. Gu, M. Jian, L. Huang, Z. Sun, A. Li, Y. Pan, J. Yang, W. Wen, W. Zhou, Y. Lin, *Nat. Nanotechnol.* **2021**, *16*, 1141-1149.

[6] F. Huang, M. Peng, Y. Chen, Z. Gao, X. Cai, J. Xie, D. Xiao, L. Jin, G. Wang, X. Wen, *ACS Catal.* **2021**, *12*, 48-57.

[7] Y. Song, S. Weng, F. Xue, A. J. McCue, L. Zheng, Y. He, J. Feng, Y. Liu, D. Li, *ACS Catal.* **2023**, *13*, 1952-1963.

[8] Y. Yue, B. Wang, C. Jin, K. Huang, Q. Zhou, R. Chang, S. Wang, Z. Pan, J. Zhao, X. Li, *ACS Catal.* **2024**, *14*, 3900-3911.

[9] F. Zhang, Y. Zhang, J. Wang, Q. Wang, H. Xu, D. Li, J. Feng, X. Duan, *Angew. Chem. Int. Ed.* **2024**, *63*, e202412637.

[10] Y. Chai, G. Wu, X. Liu, Y. Ren, W. Dai, C. Wang, Z. Xie, N. Guan, L. Li, *J. Am. Chem. Soc.* **2019**, *141*, 9920-9927.

[11] J. Ma, F. Xing, Y. Nakaya, K. i. Shimizu, S. Furukawa, *Angewandte Chemie* **2022**, *134*, e202200889.

[12] J. Ma, F. Xing, K.-i. Shimizu, S. Furukawa, *Chemical Science* **2024**, *15*, 4086-4094.

[13] X. Ge, J. Yin, Z. Ren, K. Yan, Y. Jing, Y. Cao, N. Fei, X. Liu, X. Wang, X. Zhou, *J. Am. Chem. Soc.* **2024**, *146*, 4993-5004.

[14] G. X. Pei, X. Y. Liu, A. Wang, A. F. Lee, M. A. Isaacs, L. Li, X. Pan, X. Yang, X. Wang, Z. Tai, *ACS Catal.* **2015**, *5*, 3717-3725.

[15] Y. Cao, Z. Sui, Y. Zhu, X. Zhou, D. Chen, *ACS Catal.* **2017**, *7*, 7835-7846.

[16] X. Huang, Y. Xia, Y. Cao, X. Zheng, H. Pan, J. Zhu, C. Ma, H. Wang, J. Li, R. You, *Nano Res.* **2017**, *10*, 1302-1312.

[17] G. X. Pei, X. Y. Liu, X. Yang, L. Zhang, A. Wang, L. Li, H. Wang, X. Wang, T. Zhang, *ACS Catal.* **2017**, *7*, 1491-1500.

[18] F. Huang, Y. Deng, Y. Chen, X. Cai, M. Peng, Z. Jia, P. Ren, D. Xiao, X. Wen, N. Wang, *J. Am. Chem. Soc.* **2018**, *140*, 13142-13146.

[19] W. Cheong, Y. Wang, L. Zheng, H. Xiao, C. Chen, D. Wang, Q. Peng, L. Gu, X. Han, J. Li, **2018**.

[20] Q. Feng, S. Zhao, Q. Xu, W. Chen, S. Tian, Y. Wang, W. Yan, J. Luo, D. Wang, Y. Li, *Adv. Mater.* **2019**, *31*, 1901024.

[21] Y. Liu, B. Wang, Q. Fu, W. Liu, Y. Wang, L. Gu, D. Wang, Y. Li, *Angew. Chem. Int. Ed.* **2021**, *60*, 22522-22528.

[22] F. Huang, M. Peng, Y. Chen, X. Cai, X. Qin, N. Wang, D. Xiao, L. Jin, G. Wang, X.-D. Wen, *J. Am. Chem. Soc.* **2022**, *144*, 18485-18493.

[23] Q. Gao, Z. Yan, W. Zhang, H. S. Pillai, B. Yao, W. Zang, Y. Liu, X. Han, B. Min, H. Zhou, *J. Am. Chem. Soc.* **2023**, *145*, 19961-19968.

[24] Z. Li, J. Zhang, J. Tian, K. Feng, Y. Chen, X. Li, Z. Zhang, S. Qian, B. Yang, D. Su, *ACS Catal.* **2024**, *14*, 1514-1524.

[25] C. Sui, W. Dong, M. Wang, F. Huang, S. Xiang, H. Wang, J. Chen, C. Li, M. Peng, N. Wang, *J. Am. Chem. Soc.* **2025**.

[26] J. Zhang, W. Wang, J. Chen, X. Cai, L. Yang, M. Peng, Y. Wang, Y. Si, F. Hong, X. Chen, *Angew. Chem. Int. Ed.* **2025**, *64*, e202515937.
